# Bulked Segregant Analysis and Association Analysis Identified the Polymorphisms Related to the Intermuscular Bones in Common Carp (*Cyprinus carpio*)

**DOI:** 10.3390/biology11030477

**Published:** 2022-03-21

**Authors:** Ming-Shu Cui, Ran Zhao, Qi Wang, Yan Zhang, Qing-Song Li, Mei-Di Huang Yang, Xiao-Qing Sun, Jiong-Tang Li

**Affiliations:** 1 Key Laboratory of Aquatic Genomics, Ministry of Agriculture and Rural Affairs, Chinese Academy of Fishery Sciences, Chinese Academy of Agricultural Sciences, Beijing 100141, China; cms851860@163.com (M.-S.C.); zhaoran@cafs.ac.cn (R.Z.); wangqi@cafs.ac.cn (Q.W.); zhangy@cafs.ac.cn (Y.Z.); liqingsong686@163.com (Q.-S.L.); huangymd@163.com (M.-D.H.Y.); sunxiaoqing@cafs.ac.cn (X.-Q.S.); 2 Beijing Key Laboratory of Fishery Biotechnology, Beijing 100141, China; 3 National Demonstration Center for Experimental Fisheries Science Education, Shanghai Ocean University, Shanghai 201306, China

**Keywords:** intermuscular bone, bulked segregant analysis, SNP, association analysis, joint effect, common carp

## Abstract

**Simple Summary:**

Many widely cultured freshwater fish species, such as common carp, belong to the Cyprinidae family. However, most cyprinids have numerous and complex intermuscular bones (IBs), resulting in an adverse effect on cyprinid fish meat processing and consumption. Numerous studies have been trying to understand the development mechanism of IBs and to identify the SNPs associated with the total IB number. However, the SNPs associated with different forms of IBs have been studied less thoroughly. The joint effects of the SNPs on IB development also remain poorly understood. The common carp has numerous geographical populations and domesticated strains, diversifying its phenotypes. The question of whether consensus IB-related SNPs or genes exist among multiple strains of common carp has also not yet been answered. Selective breeding of IB-reduced common carp has been hindered due to a lack of effective molecular markers. To answer these questions, we performed bulked segregant analysis (BSA) to detect the consensus SNPs in three strains. The consensus BSA-SNPs and the other SNPs in their flanking regions were validated in additional individuals. The SNPs associated with the frequency of different IB types were identified. We examined the joint effects of significant SNPs on the numbers of different types of IBs. The identified genetic markers may benefit future selective breeding and reduce the IB number in common carp.

**Abstract:**

The allotetraploid common carp is one of the most important freshwater food fish. However, the IBs found in allotetraploid common carp increase the difficulty in fish meat processing and consumption. Although candidate genes associated with the total IB number have been identified, the SNPs associated with the numbers of the total IBs and different forms of IBs have not yet been identified, hindering the breeding of IB-reduced common carp. Herein, the numbers of different types of IBs in three common carp strains were measured. Using whole-genome resequencing and bulked segregant analysis in three pairs of IB-more and IB-less groups, we identified the consensus nonsynonymous SNPs in three strains of common carp. Screening the flanking regions of these SNPs led to the detection of other SNPs. Association study detected 21 SNPs significantly associated with the number of total IBs, epineural-IBs, and ten detailed types of IBs. We observed the joint effects of multiple SNPs on each associated IB number with an improved explained percentage of phenotypic variation. The resulting dataset provides a resource to understand the molecular mechanisms of IB development in different common carp strains. These SNPs are potential markers for future selection to generate IB-reduced common carp.

## 1. Introduction

Most cyprinids have numerous and complex intermuscular bones (IBs) [1], which are embedded into the myosepta [2]. The IBs were classified based on their locations into the epineural bones (en-IBs), epipleural IBs (ep-IBs), and epicentral IBs (ec-IBs) [3]. The IBs were also sorted based on their morphology into seven forms, including I type, Y type, one-end-unequal-bi-fork type (OEUBF), one-end-multi-fork type (OEMF), two-end-bi-fork type (TEBF), two-end-multi-fork type (TEMF), and tree-branch type (TB) [4]. The IBs increase the labor required to process cyprinids meat, hindering the meat consumption. Therefore, to reduce the IB count in cyprinids and understand the mechanisms of IB development, comparative genomic, transcriptomic, and proteomic analyses were performed to identify the genes and pathways associated with IB development [5,6,7]. Numerous protein-coding genes [8,9,10] and noncoding RNAs [6] have been found to be involved in IB development in various fishes, suggesting the complex regulation of IB development. Some genes were knocked out by using the clustered regularly interspaced short palindromic repeats (CRISPR)-Cas9 technology, decreasing the IB count in fish [11]. Although genome editing provides promise of generating the IB-free or IB-reduced fish, the edited fish exhibited developmental defects [12,13]. Generating IB-deficient fish without any defects by genetic breeding or ploidy breeding may be more effective [4,14]. However, most current research focused on the identification of SNPs associated with the number of total IBs. The SNPs associated with different forms of IBs have been studied less thoroughly. Analyzing multiple SNPs is a promising approach to finding genetic effects beyond single-locus associations. Nevertheless, the joint effects of the SNPs on IB development remain poorly understood.

Common carp, an allotetraploid fish [15], is cultured worldwide. In China, common carp accounts for about 11.2% of annual freshwater fish production. Over 20 strains of common carp have been bred and cultivated in China. In addition to serving as a food fish, it is also a common ornamental fish species [16]. Research on common carp IB development is ongoing. Quantitative trait locus (QTL) analysis and comparative transcriptomic study have been used to analyze candidate genes associated with the total IB count in common carp [17,18]. However, the SNPs associated with the numbers of the total IBs and different forms of IBs have not been identified yet. Common carp has numerous geographical populations and domesticated strains, diversifying the phenotypes [19]. Whether consensus IB-associated SNPs or genes exist among multiple strains of common carp also requires further study. Finally, our recent study revealed that two subgenomes in the allotetraploid common carp underwent the differential expression balance in response to different conditions, dampening the stimulus’ impact on the homoeologous expression [15]. Whether both homoeologous subgenomes participate in IB development is still unknown.

Herein, we performed bulked segregant analysis (BSA) to detect SNPs in each of three strain pairs consisting of IB-less and IB-more bulks. The consensus BSA-SNPs and their neighboring SNPs among three strain pairs were identified in additional individuals. Then the SNPs associated with the numbers of different types of IBs were identified. We examined the joint effects of significant SNPs on IB numbers. Our study advanced knowledge in this field, allowing a better understanding of the genetic diversity of IB numbers in common carp, and provided genetic markers for future selection to reduce the IB number in common carp.

## 2. Materials and Methods

### 2.1. Ethics Statement

This study was performed under the recommendations on animal care and use for scientific purposes established by the Animal Care and Use Committee of the Chinese Academy of Fishery Sciences (ACUC-CAFS). All the fish in this study were euthanized with MS222 solution (a concentration of 40 mg/L).

### 2.2. Counting and Classifying the IBs

In November 2020, we collected the six-month-old juvenile common carp including the variety Hebao (HB, Wuyuan, China), the variety Wenqing (WQ, Zhaoqing, China), and the variety Hehua (HH, Quanzhou, China) (Supplementary Figure S1). The samples had been cultivated in one pond together at the experimental fish station of the Chinese Academy of Fishery Sciences (Fangshan, Beijing, China) for half a year. The fish were fed with the same commercial diet (Tongwei, China) in the pond. In total, 122 common carp individuals were randomly selected, including 38 HH individuals, 50 HB individuals, and 34 WQ individuals.

Before counting the IB number, the back muscles were dissected from each individual. We followed the method of Lv et al. [20] to separate the IBs from the muscle. The IBs were photographed in order with a digital camera. The IBs were first classified into two major types:epineural bones (en-IBs) and epipleural bones (ep-IBs). The en-IBs and ep-IBs were further sorted into seven types: I type, Y type, OEUBF, OEMF, TEBF, TEMF, and TB (Supplementary Figure S2). The numbers of total IBs, en-IBs, ep-IBs, and 14 types of IBs were applied to the association study.

We investigated the phenotypic distances among three strains using principal component analysis (PCA) with the ‘pcrcomp’ function in R package with the above 14 types of IBs. The first three eigenvectors were plotted. The Spearman correlation between any two traits across the 122 individuals was calculated with the ‘correl’ function in R. The 14 types of IBs were clustered on the basis of the correlations using the ‘single method’ and visualized using the R function ‘heatmap’.

### 2.3. Genome Resequencing and Bulk Segregant Analysis

Genomic DNA from the back muscle of each individual was extracted using a commercial DNA extraction kit (TIANGEN Biotech, Beijing, China), which was based on the traditional phenol-chloroform extraction method. The DNA was treated with RNase and then stored at −20 °C until further analysis. The DNA quality was checked using gel electrophoresis and the quantity was measured with a UV spectrophotometer, NanoVue Plus (GE healthcare, Boston, MA, USA). In each strain, seven samples with the fewest total IBs were clustered together as an IB-fewer group (F-pool) and another seven samples with the most total IBs as an IB-more group (M-pool). DNA from each group with a concentration of at least 100 ng/ul was used to construct a paired-end genome library, which was sequenced on an Illumina Novaseq 6000 (Illumina, San Diego, CA, USA) platform with the 150-bp PE mode and coverage of at least 37-fold.

The raw reads of each group were filtered using Trimmomatic v0.35 [21] and mapped to the latest common carp reference genome (GenBank no: GCA_018340385.1 [15]) using BWA v0.7.17 [22]. Picard in the Genome Analysis Tool Kit (GATK, v3.8) [23] was used to filter out the low-quality alignments and PCR-duplicated fragments. Variants were called using HaplotypeCaller with the parameters of ‘-ERC GVCF -stand-call-conf 20-mbq 20′ and then GenotypeGVCFs with default parameters. The SNPs were identified using SelectVariants with the parameters of ‘-select-type SNP’ and filtered using VariantFiltration with the parameters of ‘-cluster 4—window 10—mask-extension 3—filter-name LowQual—filter QUAL <20′ in GATK. In each strain, to identify the SNP with different frequencies between two groups, the VCF files from the IB-fewer (F-pool) and IB-more (M-pool) groups were inputted to MutMap [24] with the minimum base quality of 20 and 5000 simulation replicates. An SNP-index of one SNP in each pool was represented as the proportion of reads harboring it. The Δ(SNP-index) of this SNP was the difference between the SNP-index in the M-pool and that in the F-pool. If the Δ(SNP-index) was over the 95% confidence interval of all Δ(SNP-index), this SNP was considered as M-pool-enriched. Likewise, we identified the F-pool-enriched SNPs. The SNPs identified by the BSA analysis were designated as the BSA-SNPs.

In each strain, we examined the genomic locations and putative protein-coding changes by these BSA-SNPs using the package ANNOVAR version 2020-06-07 [25]. Based on the common carp gene annotation, the genomic locations of these BSA-SNPs were categorized into the exonic regions, splicing sites, 5’ untranslated regions (UTRs), 3’ UTRs, intronic regions, upstream and downstream regions, and intergenic regions. The protein-coding changes affected by the exonic SNPs were further classified into synonymous mutation, nonsynonymous mutation, stop-gain, and stop-loss. The latter three types of SNPs were attributed to deleterious mutations. For each strain, we detected the Gene Ontology (GO) terms overrepresented in the genes with the deleterious exonic BSA-SNPs using TBtools v1.075 [26] with *p* values < 5%. This software performs the GO term enrichment analysis based on hypergeometric distribution.

We investigated whether consensus SNPs would be observed in all three strains. The consensus SNPs were also categorized using ANNOVAR [25]. We only found two types of exonic SNPs, including synonymous SNPs (sSNPs) and nonsynonymous SNPs (nsSNPs). The nsSNPs were retained in the association study of IB traits.

### 2.4. Amplification, SNP Calling, and Genotyping

The specific primers for four BSA-nsSNPs were designed to amplify the loci and their flanking regions of 500 bp in 122 individuals (Supplementary Table S1). The PCR amplifications were performed in a volume of 50 µL containing 1 µL of DNA template, 2 µL of each primer (10 umol), 25 µL of easyTaq PCR SuperMix (TransGen Biotech, Beijing, China), and 20 µL of nuclease-free water. The amplification profile included 3 min at 94 °C, 40 cycles of 30 s at 94 °C, 30 s at the annealing temperature of 55 °C, 40 s at 72 °C, and a final extension of 10 min at 72 °C. The amplification products were purified with a commercial DNA fragment purification kit (Taihegene, Beiing, China) and then sequenced using the the Sanger method. Since the amplified region of each SNP was, at most, 1 kb, two ends of one round of Sanger sequencing covered the complete region.

Since the protein-coding genes in the common carp A and B subgenomes have high nucleotide similarities [15], to avoid misalignments and putative artificial SNPs, all amplified sequences were mapped to the genome assembly using blastn analysis with an e-value of 10^−5^. If the best aligned region of each sequence was not the expected region, this sequence was discarded.

Before genotyping, we identified other neighboring non-BSA-SNPs around the BSA-SNPs. The retained amplified sequences were aligned to their corresponding reference genomic regions with novoSNP [27], with the reliable region ranging from 50 bp to 650 bp. In each individual, if one locus had up to two sequencing peaks and an F-score > 30, it was reserved. Those sites in one sample having at least three peaks, possibly resulting from the sequencing errors, were not included in the analysis. We identified the high-quality non-BSA-SNPs and heterozygote/homozygote following Zhang et al.’s protocol [28]. In brief, a homozygote had only one sequencing peak and a heterozygote had two sequencing peaks. The effects of non-BSA-SNPs on the coding sequences were also classified into synonymous substitution, nonsynonymous substitution, stop-gain, and stop-loss.

### 2.5. Examining the Genetic Diversities of the Examined SNPs

If one genotype of this locus was observed in fewer than four samples, this genotype was not included in the genetic diversity analysis. We calculated the genetic distances and population structures among three strains with all retained genotypes of the BSA-SNP and non-BSA-SNP loci. The genetic distances were estimated using principal component analysis (PCA) in Tassel 5.0 [29]. The first two eigenvectors were plotted. With the genotypes of each locus, the population structure among three strains was analyzed using Admixture 1.3.0 [30]. K was set from 2 to 6. The population structures and the genetic compositions of each individual were displayed with pophelper v2.3.1 [31]. As three strains were grouped together in the PCA analysis and had similar structures, we combined these strains into one population. If one SNP was successfully genotyped in at least 70 samples, we measured its genetic diversity with four indicators, including the observed heterozygosity (Ho), expected heterozygosity (He), polymorphism information content (PIC), and minor allele frequencies (MAF), using Genepop [32]. Mann-Whitney U tests were used to examine the genetic diversity distributions among three gene loci.

### 2.6. Identifying the SNPs Associated with the Numbers of IBs

Associations between the numbers of each type of IBs and the genotypes among all individuals were analyzed using Tassel 5 [29], with the general linear model (GLM) considering the PCA-matrix, which had the top five principal components. A test of 100,000 permutations was performed. The GLM model was widely used in the association study between the polymorphisms and the growth traits of common carp, including body weight and body length [33,34,35,36]. The percentage of phenotypic variation (PV) explained by each SNP was represented as Marker R^2^, which was calculated with Tassel 5. We also performed the association study for each IB trait using analysis of variance (ANOVA). For the count of each type of IB, we grouped the individuals on the basis of their genotypes of one SNP. A pairwise comparison of the counts among the genotypes of this SNP with ANOVA was performed. If a significant difference in the counts between two compared groups was found with a *p* value < 0.05, this SNP was significantly related to the number of this IB type with ANOVA. For each type of IB, one SNP was considered significantly associated with the count if it had a *p* value < 0.05 in the GLM model and a *p* value < 0.05 in the ANOVA.

### 2.7. Joint Effects of Significant SNPs on the IB Numbers

Analyzing the joint effects of multiple SNPs on the phenotype is a promising approach to finding genetic effects beyond single-locus associations [37]. Hence, we performed the joint analysis of multiple SNPs to detect a larger effect on the numbers of IBs than that of the individual SNP. The analysis also helped to detect the genotype combination associated with fewer IBs. To estimate the joint effects of the significantly associated SNPs on the number of each type of IB, all SNP loci associated with this type of IB were selected, and we generated different combinations of genotypes from all of these SNPs. Only the genotype combinations observed in at least three individuals were retained. We compared the IB numbers among all genotype combinations with ANOVA. For each type of IB, the percentages of PV explained by the SNP combination were estimated with the function ‘lm’ [38] in the R package.

## 3. Results

### 3.1. Comparison of the IB Numbers in Different Strains

**Figure 1:**
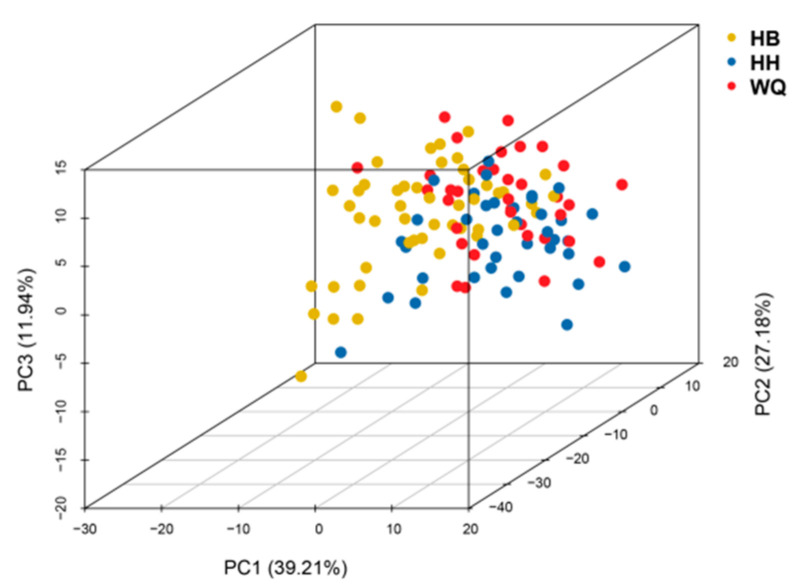
The PCA plot clustering three common carp strains with the numbers of 14 types of IBs.

The numbers of total IBs in the HB, HH, and WQ strains fell within the range of 80–108 (median = 99), 79–101 (median = 95), and 76–104 (median = 95), respectively (Supplementary Table S2). PCA analysis based on the numbers of 14 types of IBs grouped these three strains together (Figure 1). The first two principal components explained 39.21% and 27.18% of the total genetic variance, respectively. The third component accounted for 11.94% of the variances.

In all strains, we did not observe the epicentral IBs. The en-IBs (median = 66) were the dominant type of IBs compared with the ep-IBs (median = 30.5). Among the 14 different types of IBs, the en-I and ep-I were the two main types of IBs (both median = 20), followed by the en-Y IBs (median = 16). We did not observe the ep-TB IBs in all samples and found only one sample with the en-TB IBs.

**Figure 2:**
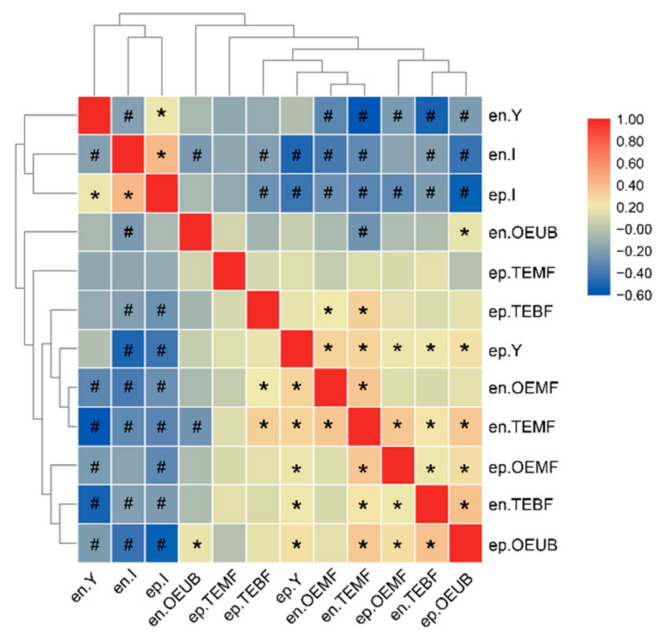
Heat map showing count correlations among 14 types of IBs. * two compared traits are significantly positively correlated. # two compared traits are significantly negatively correlated.

Excluding the ep-TB and en-TB IBs, we clustered the other 12 types of IBs into two groups based on a matrix of pairwise number comparisons between each of the two forms of IBs (Figure 2). The first group consisted of the en-Y, en-I, and ep-I IBs, which were the dominant types of IBs. The en-I and ep-I IBs were significantly positively correlated, while they were significantly negatively correlated with the en-Y IBs. The second group included the other nine types of IBs, the numbers of which were lower than the first group. Most members in this group were significantly positively correlated with one another, except for the en-OEUB and ep-TEMF IBs. We also observed that the number of total IBs had a significantly positive correlation with the numbers of en-IBs and ep-IBs (Pearson’s correlations = 0.822 and 0.852, *p* < 0.05, respectively, Supplementary Figure S3), suggesting that the number of total IBs was affected by both numbers of en-IBs and ep-IBs.

### 3.2. Identification of Segregant SNPs by BSA

In each strain, we sorted the samples with the fewest IBs and the most IBs into two extreme phenotypic groups, respectively. In the HB strain, the IB-fewer and IB-more groups had total IB number means of 87.3 ± 4.29 and 104.5 ± 1.99, respectively, with a ratio of 1.197 (Supplementary Figure S4 and Table S2). The IB mean ratios of two extreme groups in the other two strains were 1.16 (HH strain) and 1.17 (WQ strain), respectively. We sequenced six pools of the IB-fewer and IB-more fish in three strains and obtained about 60 Gb of sequence data with approximately 37-fold depth (ranging from 35.9–41.8×) per pool. Over 98.8% of these reads were aligned to the common carp genome (Table 1).

We obtained a basic set of 20,718,699 SNPs (1.23% of genome size, one SNP per 81.1 bp), including 12,531,928 SNPs in the HH strain, 10,835,374 SNPs in the HB strain, and 11,477,507 SNPs in the WQ strain (Supplementary Figure S5). In the basic set, the ratio of transition to transversion (Ts/Tv) was 2.2. The C/T transition accounted for 34.4% of all SNPs. A total of 87.5% of SNPs were located in the intergenic regions or intronic regions, and 3.7% were exonic (Supplementary Figure S6a). The percentage of sSNPs (61.4%) was higher than that of nsSNPs (38.1%) (Supplementary Figure S6b). A core set of 4,812,113 SNPs was shared among the three strains. In this core SNP set, the Ts/Tv ratio (2.25), C/T transition proportion (34.6%), genomic distribution of SNPs, and percentage of sSNPs were almost equivalent to those in the basic set (Supplementary Figure S7). The genomic distribution of SNPs and the percentages of different types of exonic SNPs in each strain also had similar distributions to those in the basic and core SNP sets (Supplementary Figures S8 and S9).

The BSA analysis identified 114,909 significant segregant SNPs in the HB strain, 118,240 SNPs in the HH strain, and 230,667 sites in the WQ strain, respectively (Supplementary Table S3). In each strain, the proportion of segregant SNPs accounted for 0.9–2.0% of all SNPs. The genomic and exonic distributions of the BSA-SNPs were subject to those of all SNPs in each strain (Supplementary Figures S10 and S11). The Ts/Tv ratio and C/T transition proportion of the BSA-SNPs were slightly higher than those of all SNPs (Supplementary Table S4). In each strain, 4.2–4.57% of the BSA-SNPs were exonic and the majority (59.66–59.79%) of these exonic BSA-SNPs were synonymous. The exonic BSA-SNPs were located in 6008 genes in the HB strain, 5761 genes in the HH strain, and 7073 genes in the WQ strain (Supplementary Figure S12). Most genes with the exonic BSA-SNPs were strain-specific. The Venn diagram showed that only 596 genes with the exonic BSA-SNPs occurred in three strains.

In the HB strain, 2307 genes with the deleterious exonic BSA-SNPs were enriched in 400 GO terms (Supplementary Figure S13 and Table S5). A total of 2192 genes in the HH strain were enriched in 330 GO terms, and in the WQ strain, 2676 genes with the satisfied exonic BSA-SNPs were enriched in 393 GO terms. In total, 22 GO terms were covered in three strains.

A deeper investigation found 207 BSA-SNPs shared by all three strains (Supplementary Figure S14), suggesting that most BSA-SNPs were strain-specific. They were distributed close to or in 318 genes (Supplementary Table S6). Most of them (193) were located in the intergenic or intronic regions (Table 2). Only eight SNPs, including four sSNPs and four nsSNPs, were located in the coding exons of eight genes. Four nsSNPs were encoded in four genes, which were in the chromosomes of A1, A16, A18, and B25, respectively. The first nsSNP in the A1 chromosome was found in the exon of *Mtap*, an enzyme converting the phosphorylation of S-methyl-5′-thioadenosine to adenine and 5-methylthioribose-1-phosphate [39]. The second nsSNP in the A16 chromosome belonged to the *Samd3* (sterile alpha motif domain-containing protein 3) gene [40]. The third nsSNP in the A18 chromosome was located in the *Ripor1* gene (also named FAM65A), Rho family-interacting cell polarization regulator 1. The final nsSNP identified in the B25 chromosome was detected in the *Neogenin* gene, a receptor for bone morphogenetic proteins [41].

### 3.3. Genetic Diversity of Three Sequenced BSA-SNP Regions

Four exonic ns-BSA-SNPs in four genes observed in all three strains were genotyped for the association studies. Due to the assay capacity and the limitations of primer design for the genotyping analysis, the ns-BSA-SNP in *Ripor1* was not amplified. The other three ns-BSA-SNPs were successfully genotyped. Among the 122 samples, the A1:3023753 locus (*Mtap*) was successfully sequenced in 120 samples with the A16:2710463 site (*Samd3*) in 93 samples and the B25:3800828 locus (*Neogenin*) in 101 samples. Except for the ns-BSA-SNP in *Samd3*, the polymorphisms of two ns-BSA-SNP loci in *Mtap* and *Neogenin* were successfully validated. Besides the ns-BSA-SNPs, we also called another 47 non-BSA SNPs neighboring these loci (Supplementary Table S7). In the reliable amplified region of *Mtap* with a length of 516 bp, we detected 17 SNPs with a polymorphism rate of 3.29%. In the A16 region with a size of 564 bp, we called only five SNPs with a polymorphism rate of 0.89%. Another 27 SNPs were identified in *Neogenin* with a higher polymorphism rate of 3.95%.

A total of 25 SNP loci had only two genotypes with 23 loci and 1 locus having three and four genotypes, respectively (Supplementary Table S8). Among the 49 SNPs, 25 were located in the introns and 24 were in the exons. We identified five ns-SNPs in the A1 (three SNPs) and B25 loci (two SNPs), including two BSA-SNPs and three non-BSA-SNPs. The Ho distributions in these three gene loci were not significantly different (*p* value = 0.809, 0.109, and 0.120, respectively). Likewise, the He distributions in these three gene loci were not significantly different (*p* value = 0.181, 0.283, and 0.614, respectively). PIC analysis showed that 11 SNP loci (6 and 5 in the A1 and B25 locus, respectively) displayed low polymorphism levels (PIC < 0.1) and that the remaining loci had a polymorphism rate over 0.1. The PIC values in these three gene loci were not significantly distributed (Mann–Whitney U test, *p* value = 0.197, 0.614, and 0.283, respectively). No significant MAF distribution differences among these three regions were observed (*p* value = 0.101, 0.189, and 0.579, respectively).

PCA based on all A1 genotypes clustered three common carp strains together (Supplementary Figure S15a). The first two principal components explained 25.39% and 17.82% of the total genetic variances, respectively. Similar phenomena of clustering together were observed with the genotypes of the A16 and B25 loci (Supplementary Figure S15b,c). These data proved that these three strains had a similar genetic background. In the admixture plots with different K values (K from two to six), three strains had similar genetic components of these three loci (Supplementary Figure S16). There were no strain-specific SNPs in these three loci, as evidenced by both PCA and population structure analyses. Taken together with the similar IB number distribution among the three strains, we combined these three strains into one population in the association analyses.

### 3.4. SNPs Associated with the IB Numbers

Among the 49 SNPs, 21 were identified to be significantly associated with the numbers of the total IBs, en-IBs, and ten different types of IBs using GLM and ANOVA (Table 3 and Supplementary Figure S17). The remaining types of IBs, including ep-IBs, en-TB, ep-OEUB, ep-TEMF, and ep-TB, were not found to have associated SNPs. The mean values of the latter four types of IBs were 0.01 ± 0.09, 5.60 ± 2.47, 0.25 ± 0.58, and 0 ± 0, respectively. Few IBs of these types were observed among individuals, possibly hindering the identification of the associated SNPs. Two SNPs in *Samd3* were associated with the numbers of three types of IBs (Supplementary Figure S18a–c). However, 8 SNPs in *Mtap* and 11 in *Neogenin* were the major SNPs related to IB numbers. Eight SNPs were identified to be associated with the numbers of at least two types of IBs. Except for the en-IBs, ep-I, and ep-OEMF with only one SNP, the total IBs and other eight types of IBs had at least two associated SNPs. Intriguingly, seven types of IBs had SNPs from at least two genes.

Among 17 SNPs in *Mtap*, 8 were significantly associated with the numbers of seven types of IBs, including total IBs, en-I, en-OEUB, en-TEBF, en-TEMF, en-Y, and ep-I (Supplementary Figure S18d–n). Three SNPs were identified to be associated with the numbers of two types of IBs. For the total IBs, en-OEUB, en-TEBF, and en-TEMF, the heterozygotes of the associated SNP loci were significantly associated with fewer numbers than the homozygotes. However, the trends were reversed for the en-I, en-Y, and ep-I. Most associated SNPs in this region were synonymous and only one nsSNP of *Mtap* was associated with the number of en-TEBF IBs.

Among 27 SNPs in *Neogenin*, 12 were significantly associated with the numbers of 10 types of IBs, excluding en-OEUB and ep-I (Supplementary Figure S18o–af). Two SNPs were identified to be associated with the numbers of two types of IBs and two SNPs with three types of IBs. For the total IBs, en-IBs, en-TEMF, and en-Y, the heterozygotes of the associated SNP loci were significantly associated with fewer numbers than the homozygotes. However, the trend was reversed for the en-OEMF, en-Y, ep-OEMF, and ep-TEMF. Interestingly, we observed the coexistence of dominant heterozygotes and homozygotes of *Neogenin* associated with the numbers of en-I and en-TEBF. Most associated SNPs in *Neogenin* were synonymous and only one ns-SNP of B25.3800710 was significantly related to the numbers of en-I, en-TEBF, and en-TEMF IBs.

Two SNPs in *Mtap* and *Neogenin* were found to be significantly associated with the total IB number using both GLM and ANOVA (Table 3). The homozygotes of these two loci were associated with more IBs than the heterozygotes. These two loci could explain 4.72% and 5.9% of the total IB number variation, respectively. The SNP of B25.3800532 was also significantly associated with the number of en-IBs. Likewise, the homozygote of this locus corresponded to more IBs than the heterozygote.

### 3.5. Joint Effects of Significant SNPs on the IB Numbers

The numbers of nine types of IBs had at least two associated SNPs. The SNPs in *Mtap* and *Neogenin* were significantly associated with the numbers of four types of IBs (total IBs, en-I, en-TEBF, and en-Y). The en-OEMF number had associated SNPs in *Samd3* and *Neogenin* and the en-OEUB number was associated with the SNPs in *Mtap* and *Samd3*. We also found that the SNPs in these three genes were associated with the number of en-TEMF. These data suggest that these seven types of IBs might be regulated by these genes. For nine types of IBs with at least two associated SNPs, we were interested in testing two simple joint effects that failed to be identified by single-marker analysis: (a) whether a genotype combination corresponding to the lowest IB number existed, and (b) whether the explained percentage of PV could be improved by multiple SNPs compared with a single SNP.

For the number of total IBs, we observed only three combinations in two SNP loci in the population (Supplementary Table S9 and Figure S19a), including GG/TT (both homozygotes in two SNP loci, named as hap1), GT/TT (one homozygote in A1.3023796 and one heterozygote in B25.3800532, hap2), and GG/TG (one heterozygote in A1.3023796 and one homozygote in B25.3800532, hap3). We did not find the combination of both heterozygotes in two SNP loci. The first combination was observed in 101 individuals, and was dominant in the population. The explained percentage of PV was increased from 4.72% with one SNP to 10% with two loci (Table 4).

In the other eight types of IBs, we found higher number deviations among different genotype combinations than those in the single-marker analysis (Supplementary Figure S19b–i). For the en-I number, we observed seven genotype combinations from four SNP loci. In the single-SNP analysis, the mean IB number ratio between the homozygote and the heterozygote of each single SNP ranged from 0.742 to 0.927 and the lowest mean IB number was 15.9 ± 4.26, observed in the homozygotes of B25.3800904. As shown in Table S9, these combinations had different numbers of samples where most individuals had a mean en-I number of 21.61 ± 4 (observed in the hap3 combinations). The lowest mean en-I IB number (14 ± 5.2) and the highest number (25 ± 2) corresponded to two extreme genotype combinations (hap7 and hap1, respectively), with a ratio of 0.56. The explained percentage of PV was improved from 5.04% with one SNP to 15.93% with multiple loci. For the remaining seven types of IBs, we also observed the joint effects of all associated SNPs, demonstrated by both the decreased mean IB number and the improved explained percentage of PV.

## 4. Discussions

IBs widely exist in cyprinids and have become a critical factor affecting cyprinid consumption and processing. In total, 12 different types of IBs were observed in common carp. Few en-TB and ep-TB IBs were also observed by Li et al. [4]. We clustered these 12 types of IBs into two groups. In general, the numbers of the first group were negatively correlated with those of the second group, suggesting that the former group possibly had an antagonistic effect on the second group. On the contrary, most intra-group members were significantly positively correlated with one another. These data all suggested the complexity of IB morphology in common carp.

One notable avenue to decrease the IB number is to knock out IB-related genes by using Cripsr/Cas9 technology, generating IB-reduced fish. However, gene-edited fish are still not allowed to be cultivated or marketed. Another possible strategy to generate the IB-few fish is genome selection breeding. Current studies on common carp IB development have narrowed the IB-related genomic regions down to the gene level or the QTL level [17,18]. Nevertheless, SNPs associated with the numbers of IBs have not been identified yet. Previous studies mainly focused on comparing the number of total IBs or limited types of IBs. Herein, we executed a comprehensive association with the numbers of total IBs, en-IBs, ep-IBs, and 14 other types of IBs. We observed many IB-related SNPs in all three strains. These SNPs were associated with the numbers of different types of IBs, suggesting that they might be involved in the development of different types of IBs separately. The SNP combinations had joint effects on the numbers of multiple types of IBs, supported by both the decreased mean IB number and the improved explained percentage of PV. For instance, the smallest mean numbers of en-OEMF and en-TEMF were decreased to 0.833 ± 1.33 and 0 ± 0, respectively, if considering multiple associated SNPs together, suggesting that it is possible to select the individuals having the fewest en-OEMF and en-TEMF with the SNP sets.

Many reported IB-development-related genes, including SAM and SH3 domain containing protein 1 (*Sash1*) [5], fibroblast growth factor receptors *Fgfr2* and *Fgfr3* [42], SRY-box transcription factor 9 (*Sox9*) [43], and Osteopontin (*Opn*) [44], were identified in our study (Supplementary Table S6). We identified the BSA-SNPs in the genes of the bone morphogenetic proteins (BMP) signaling pathway [45] in different strains (Supplementary Table S10). A total of 15, 10, and 13 genes involved in the BMP signaling pathway had deleterious exonic BSA-SNPs in the HB, HH, and WQ strains, respectively (Supplementary Figure S20). We performed consensus comparisons in three different levels, including SNPs, genes, and GO processes. In three strains, only eight exonic BSA-SNPs located in eight genes were covered, whereas 596 genes with the deleterious exonic SNPs were shared. These data suggested that each strain had different exonic polymorphisms in the same gene related to regulating IB development. These 596 genes might be common regulatory genes of IB generation in these three strains. We also observed that 22 GO biological processes were found in all three strains, although not all genes in these processes had deleterious mutations. One enriched category that was common in the three strains included the assembly, movement, and organization of cilium. These processes have been reported to regulate osteoblast differentiation, polarity, and alignment to reduce bone formation [46]. The cilia prompt chondrocyte differentiation and production of a cartilage matrix. Cilia are required for increased bone formation in response to mechanical forces. The primary cilium is also associated with many signaling pathways, including primary cilium-related Ca^2+^ signaling, BMP/Smad1/5/8 signaling, and Wnt signaling [47].

Our analysis provided hints that three genes had consensus nsSNPs in three strains to regulate IB development. The homologs of these three genes participate in bone development. The dysfunction of human *Mtap* homolog causes diaphyseal medullary stenosis with malignant fibrous histiocytoma [48]. Another nsSNP was located in the *Samd3* gene. Although there was no direct evidence to prove that *Samd3* was related to bone development, a deleterious mutation in *Samd9*, a homolog of *Samd3*, causes familial tumoral calcinosis [49], representing painful ulcerative lesions and severe skin and bone infections. The protein of *Neogenin* was found to bind directly with BMP2, BMP4, BMP6, and BMP7, and to negatively regulate the functions of BMPs [41]. This gene had more associated SNPs and IB types than *Mtap* and *Samd3*, suggesting that this gene might make a greater contribution to IB development. Studying the functions of these three genes would contribute to further elucidation of IB development mechanisms in common carp and other cyprininae fish.

Different from diploid fish, the allotetraploid common carp underwent a species-specific whole-genome duplication [16]. Whether both homeologues generated by whole-genome duplication participate in the IB development requires further study. In polyploid plant, Cheng et al. detected strong subgenome parallel selection linked to the domestication of the tuberous morphotypes [50]. However, our previous expression analysis across conditions in common carp revealed differential expression balance in two subgenomes, i.e., the majority of homoeologous pairs had only one differentially expressed homoeologue [50]. Therefore, it is hypothesized that two common carp subgenomes might be under unparalleled selection on the economic traits. In this study, the homologues of these gene regions were not found to have SNPs associated with IB number. This result provided evidence that two subgenomes performed flexible and unparalleled processes related to IB development and morphology.

The association analysis of the common carp IB development could be improved in the future. Firstly, two SNPs associated with the number of total IBs explained at most 10% of PV, suggesting that other SNPs that have not yet been identified could possibly exist. Secondly, the BSA analysis detected SNPs significantly associated with the numbers of nine types of IBs. The other five IB traits, including the numbers of ep-IBs, en-TB, ep-OEUB, ep-TEMF, and ep-TB, had no associated SNPs. Thirdly, whether polymorphisms exist in the promoters, miRNA binding sites, and other functional elements of these four genes will be studied in the future.

## 5. Conclusions

We classified and measured the numbers of total IBs, ep-IBs, en-IBs, and 14 other types of IBs in three common carp strains. Using BSA analysis, we detected polymorphisms of different frequencies between two pools in each strain and identified the consensus SNPs in three strains. Fine genotyping of three polymorphic regions in *Mtap*, *Samd3*, and *Neogenin* showed similar genetic backgrounds in these three regions across the three strains. Association studies on the polymorphisms of these three genes and the numbers of different IB types identified three SNPs significantly related to IB number. The joint effect analysis indicated that these SNP combinations improved the explained percentage of PV and suggested the optimal combinations related to the reduced IB numbers. These SNPs and their optimal genotype combinations will be potential markers applied in future marker-assisted selective breeding of common carp to decrease IB numbers.

## Figures and Tables

**Table 1:** Summary of genome sequencing and alignment.

Sample	Q20 (%)	Clean Read Pairs	Depth	Mapping Ratio (%)
HBF	96.83	205,678,671	37.24	99.20
HBM	96.77	211,458,150	38.28	98.88
WQF	96.90	206,924,782	37.46	99.29
WQM	96.70	198,379,639	35.92	99.20
HHF	97.02	231,095,221	41.84	99.31
HHM	97.01	228,265,661	41.33	99.27

HBF: the individuals with the fewest IBs in the ‘HB’ strain; HBM: the individuals with the most IBs in the ‘HB’ strain; HHF: the individuals with the fewest IBs in the ‘HH’ strain; HHM: the individuals with the most IBs in the ‘HH’ strain; WQF: the individuals with the fewest IBs in the ‘WQ’ strain; WQM: the individuals with the most IBs in the ‘WQ’ strain.

**Table 2:** The genomic distributions of the BSA-SNPs shared by three strains.

Genomic Location		Number
downstream		3
exonic	nonsynonymous	4
	synonymous	4
intergenic		108
intronic		85
upstream		4

**Table 3:** Association analysis of IB numbers using GLM and ANOVA.

Trait	SNP	GLM		ANOVA		
		*p* Value	Marker R^2^ (%) ^#^	MM	Mm	*p* Value
IB	A1.3023796	0.0487	4.72	96.0 ± 5.35	90.2 ± 8.79	0.0236
	B25.3800532	0.0224	5.90	96.1 ± 5.41	90.1 ± 5.87	0.0059
en-IB	B25.3800532	0.0228	5.57	66.1 ± 3.05	62.6 ± 2.64	0.0039
en-I	A1.3023490	0.0167	6.76	20.5 ± 5.45	27.2 ± 12.80	0.0022
	B25.3800904	0.0369	5.49	21.4 ± 6.22	15.9 ± 4.26	0.0213
	A1.3023694	0.0382	5.47	20.6 ± 5.90	22.2 ± 6.95	0.0208
	B25.3800710 *	0.0498	5.04	20.6 ± 5.46	27.3 ± 12.58	0.0055
en-OEMF	B25.3800526	0.0019	7.51	3.1 ± 2.94	6.00 ± 3.44	0.0014
	B25.3800904	0.00001	16.39	3.1 ± 2.80	8.6 ± 2.07	0.00002
	B25.3800999	0.0059	8.02	2.9 ± 2.40	4.5 ± 3.72	0.0342
	A16.2710728	0.0297	5.65	4.6 ± 3.50	3.0 ± 2.85	0.0418
en-OEUB	A1.3023466	0.0221	6.26	11.4 ± 4.26	8.3 ± 2.80	0.0126
	A16.2710488	0.0450	5.13	9.9 ± 3.15	11.9 ± 4.57	0.0301
en-TEBF	A1.3023753 *	0.0333	4.80	7.9 ± 3.89	7.7 ± 3.87	0.0005
	A1.3023814	0.0414	2.96	9.7 ± 4.57	6.9 ± 3.78	0.0014
	B25.3800710 *	0.0425	4.47	8.7 ± 4.39	5.1 ± 5.79	0.0433
	B25.3800929	0.0206	3.79	8.7 ± 4.50	13.0 ± 4.12	0.0401
	A1.3023730	0.0358	4.66	9.0 ± 4.35	5.3 ± 3.13	0.0469
	B25.3801033	0.0268	5.06	8.5 ± 4.49	8.7 ± 3.95	0.0148
en-TEMF	A1.3023814	0.0138	4.64	6.9 ± 6.57	3.2 ± 3.68	0.0013
	A16.2710488	0.0183	6.09	4.6 ± 5.79	7.7 ± 6.37	0.0198
	B25.3800685	0.0276	5.48	5.8 ± 5.99	3.0 ± 4.07	0.0488
	B25.3800710 *	0.0349	5.14	5.6 ± 5.85	1.00 ± 1.91	0.0414
en-Y	A1.3023447	0.00001	15.84	14.8 ± 6.67	19.2 ± 7.38	0.0013
	A1.3023694	0.0155	6.02	14.7 ± 6.92	16.4 ± 7.49	0.0395
	B25.3801016	0.0330	6.32	15.5 ± 6.63	19.8 ± 7.42	0.0137
ep-I	A1.3023730	0.0302	5.22	19.2 ± 3.81	22.0 ± 2.14	0.0177
ep-OEMF	B25.3801033	0.0165	4.65	0.4 ± 0.72	0.9 ± 1.19	0.0357
ep-TEMF	B25.3800904	0.0149	7.02	0.2 ± 0.53	0.9 ± 1.07	0.0047
	B25.3800911	0.0380	3.64	0.2 ± 0.40	0.5 ± 0.76	0.0118
ep-Y	B25.3801016	0.0399	4.87	3.6 ± 1.62	2.1 ± 1.37	0.0073
	B25.3801028	0.0306	3.55	3.7 ± 1.47	1.9 ± 1.37	0.0019

* This SNP was nonsynonymous. ^#^ percentage of phenotypic variation explained by markers in GLM method was represented as Marker R^2^; M: major allele; m: minor allele. The data are presented as the mean ± SE representing the IB numbers in one genotype group. The SNPs marked with the asterisks are nonsynonymous.

**Table 4:** The explained percentage of PV of each type of IBs by genotype combination.

Trait	SNP	Each SNP Marker R^2^ (%)	Marker R^2^ of Genotype Combination (%)
IB	A1.3023796	4.72	10
	B25.3800532	5.90	
en-I	A1.3023490	6.76	15.9
	B25.3800904	5.49	
	A1.3023694	5.47	
	B25.3800710 *	5.04	
en-OEMF	B25.3800526	7.51	34.3
	B25.3800904	16.39	
	B25.3800999	8.02	
	A16.2710728	5.65	
en-OEUB	A1.3023466	6.26	7.44
	A16.2710488	5.13	
en-TEBF	A1.3023753 *	4.80	42.07
	A1.3023814	2.96	
	B25.3800710 *	4.47	
	B25.3800929	3.79	
	A1.3023730	4.66	
	B25.3801033	5.06	
en-TEMF	A1.3023814	4.64	17.57
	A16.2710488	6.09	
	B25.3800685	5.48	
	B25.3800710 *	5.14	
en-Y	A1.3023447	15.84	9.99
	A1.3023694	6.02	
	B25.3801016	6.32	
ep-TEMF	B25.3800904	7.02	7.95
	B25.3800911	3.64	
ep-Y	B25.3801016	4.87	18.21
	B25.3801028	3.55	

* This SNP was nonsynonymous.

## Data Availability

The genome resequencing data of six common carp stocks were deposited in the SRA database (project number PRJNA751998), respectively. The other analysis data presented in this study are available in Supplementary Materials.

## Supplementary Materials

The following supporting information can be downloaded at: https://www.mdpi.com/article/10.3390/biology11030477/s1, Figure S1: the photos of the studied common carp strains; Figure S2: illustration of the seven types of IBs observed in common carp; Figure S3: the correlation matrix among the numbers of IB, en-IB, and ep-IB; Figure S4: illustration of the IBs in the six sequenced groups of common carp types; Figure S5: the Venn diagram of identified SNPs among three strains; Figure S6: the genomic and exonic distribution of the basic SNP set; Figure S7: the genomic and exonic distribution of the core SNP set; Figure S8: the function effects of the exonic SNPs in each strain; Figure S9: the function effects of the exonic SNPs in each strain; Figure S10: the genomic distributions of the identified BSA-SNPs in three strains; Figure S11: the function effects of the exonic BSA-SNPs in three strains; Figure S12: the Venn diagram of the shared BSA-genes in three strains; Figure S13: the Venn diagram of the shared GO terms enriched by the genes with the deleterious exonic BSA-SNPs in three strains; Figure S14: the Venn diagram of the shared BSA-SNPs in three strains; Figure S15: PCA plots detected with the SNPs in three regions, respectively; Figure S16: population structures detected with the SNPs in three regions, respectively; Figure S17: the Manhattan plots showing the SNPs and their associated IB numbers; Figure S18: the homozygote and heterozygote of each SNP and corresponding IB numbers; Figure S19: the genotype combinations and corresponding IB numbers; Figure S20: the BMP-related genes identified with BSA in each strain; Table S1: the primers used for amplification of the BSA-SNP regions; Table S2: the IB numbers in 122 common carp individuals from three strains; Table S3: the identified BSA-SNPs in each strain; Table S4: slight higher Ts/Tv ratio and C/T transition in the BSA-SNPs than all SNPs in each strain; Table S5: the enriched GO terms by the genes with the deleterious exonic BSA-SNPs in three strains; Table S6: the functional annotations of 318 genes neighboring the shared 207 BSA-SNPs in three strains; Table S7: the genotypes of three loci in 122 common carp individuals from three strains; Table S8: genetic diversities of three sequenced regions; Table S9: the joint effects of the associated SNPs; Table S10: the BMP-related genes having the deleterious exonic BSA-SNPs in each strain.
