# Supplementary material for: Bulked Segregant Analysis and Association Analysis Identified the Polymorphisms Related to the Intermuscular Bones in Common Carp (Cyprinus carpio)
Source: Biology (Basel). 2022 Mar 21;11(3):477. doi: 10.3390/biology11030477 (PMC8945855; doi:10.3390/biology11030477)
Supplement: Supplementary file 1 [file biology-11-00477-s001.zip › Supplementary information.pdf]

Supplementary Figures

Figure S1. The photos of the studied common carp strains.

(a)

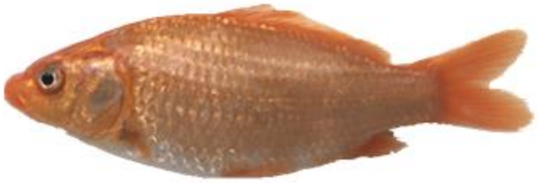

(b)

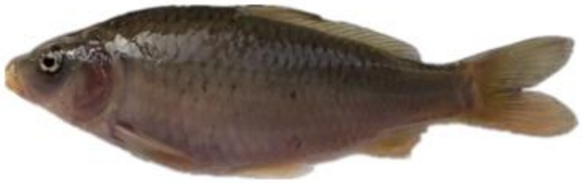

(c)

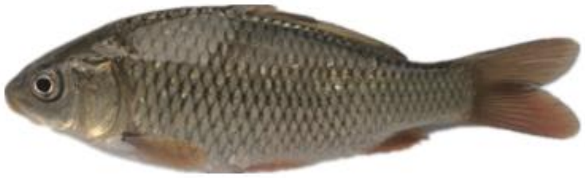

(a) var. Hebao (HB); (b) var. Hehua (HH); (c) var. Wenqing (WQ).

Figure S2. Illustration of the seven types of inter-muscular bone observed in common carp.

(a) I shape (not-fork type)

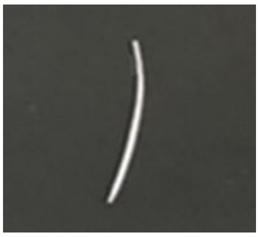

(b) 卜 shape (one-end-unequal-bi-fork type, OEUBF type)

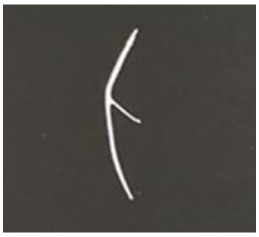

(c) Y shape (one-end-equal-bi-fork type, Y shape)

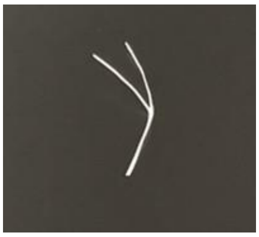

(d) one-end-multi-fork shape (OEMF shape)

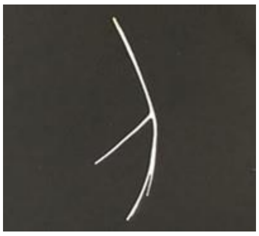

(e) two-end-bi-fork type (TEBF shape)

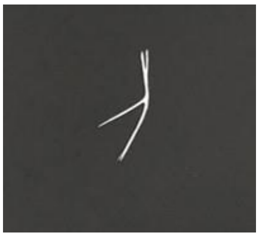

(f) two-end-multi-fork type (TEMF shape)

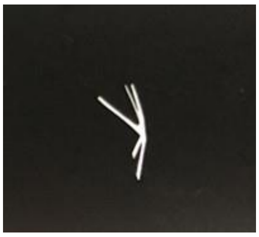

(g) tree-branch type (TB shape)

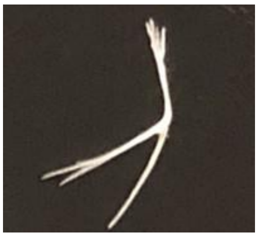

Figure S3. The correlation matrix among the numbers of IB, en-IB, and ep-IB.

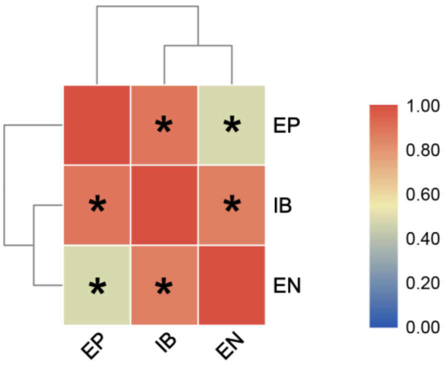

The asterisk indicats the significant correlation between two traits.

Figure S4. Illustration of the IBs in the six sequenced groups of common carp types.

(a). HH individual having the fewest IBs. **(b).** HH individual having the most IBs. **(c)**. HB individual having the fewest IBs. **(d)**. HB individual having the most IBs. (**e**). WQ individual having the fewest IBs. (**f**). WQ individual having the most IBs.

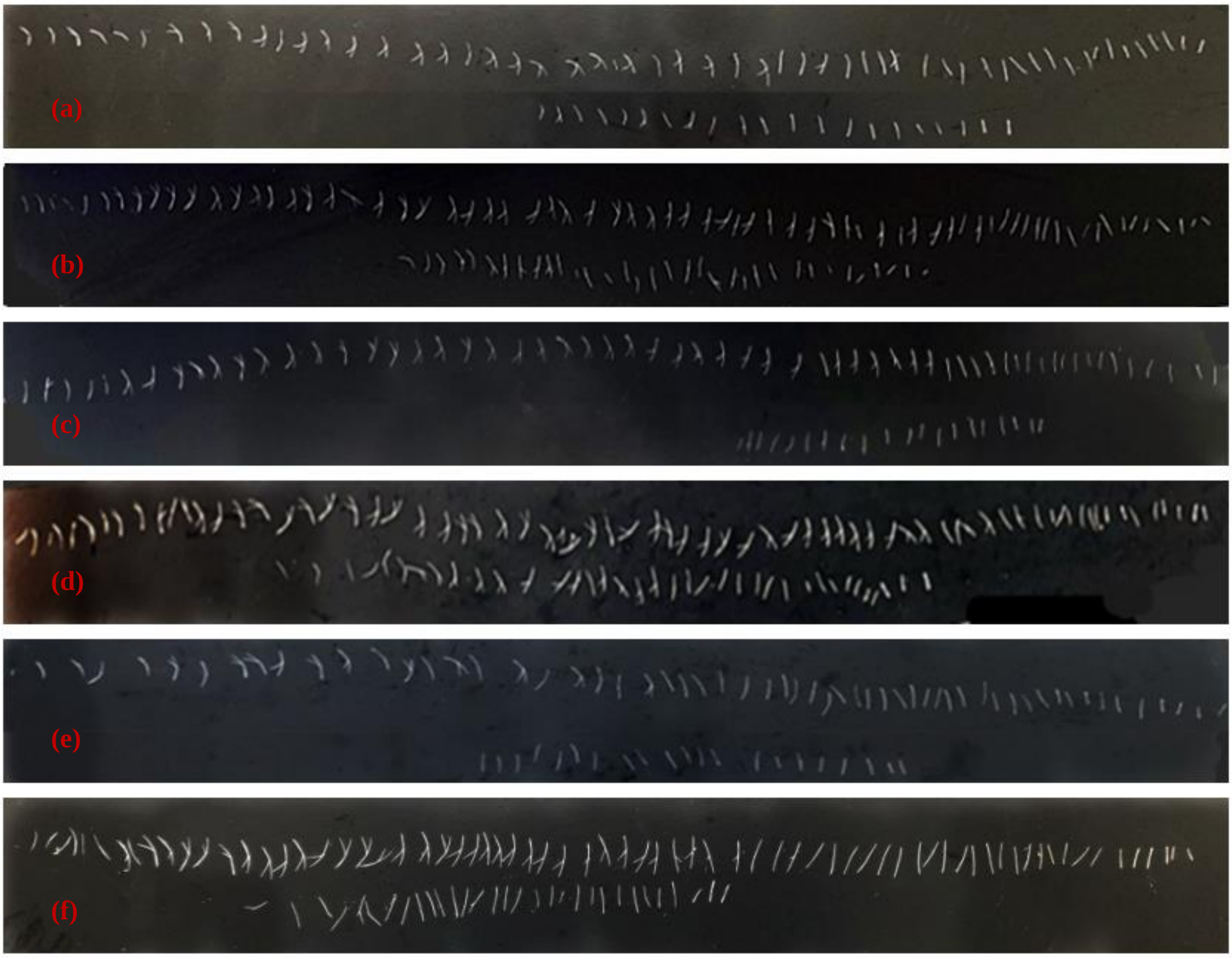

Figure S5. The Venn diagram of identified SNPs among three strains.

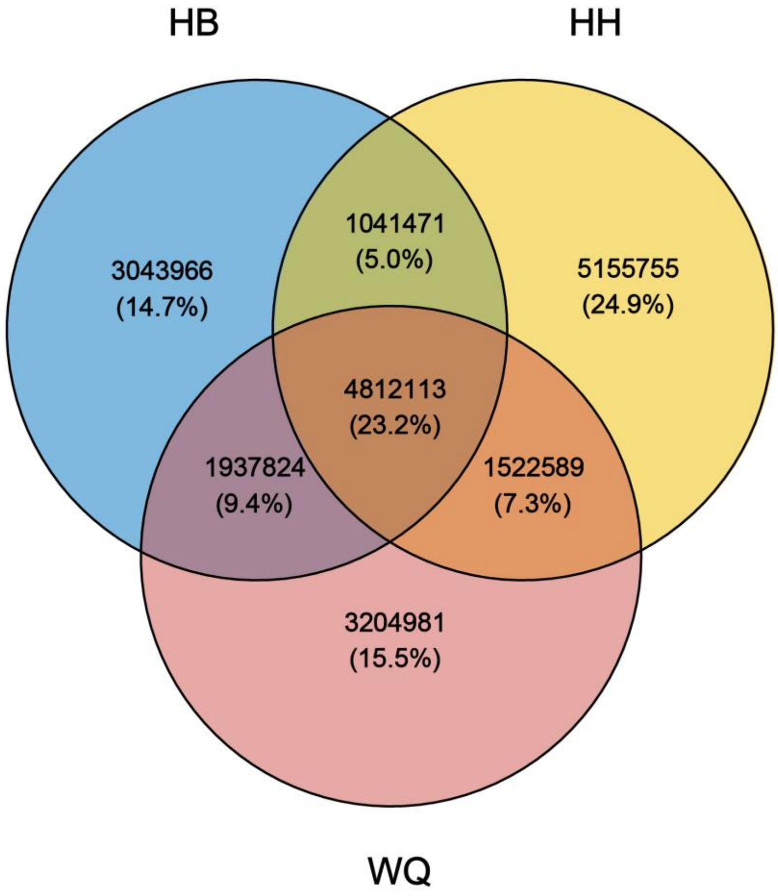

Figure S6. The genomic and exonic distribution of the basic SNP set.

(a)

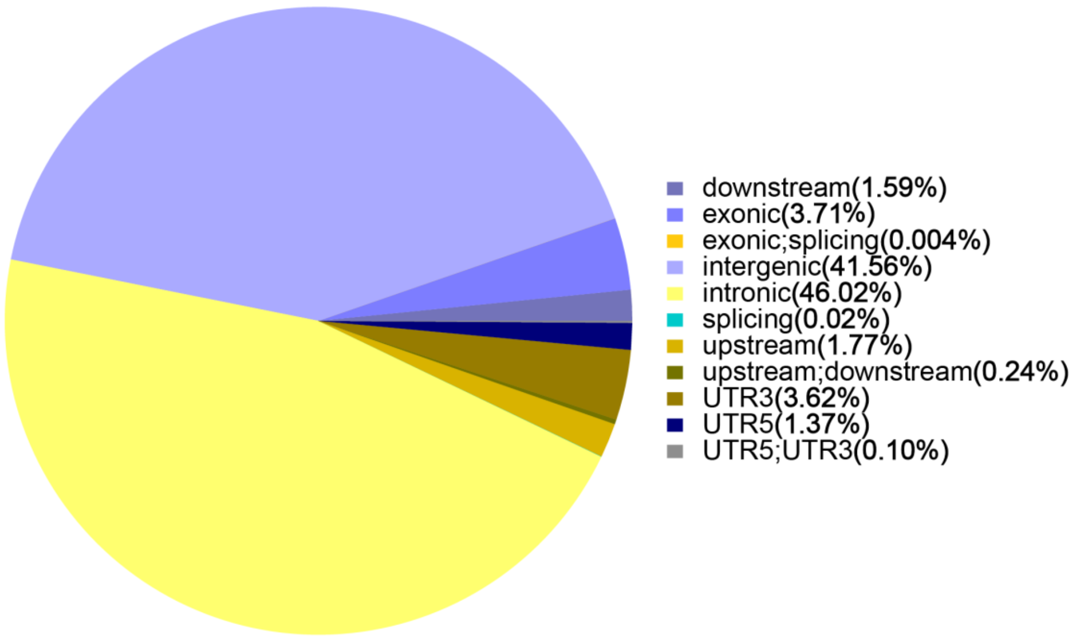

(b)

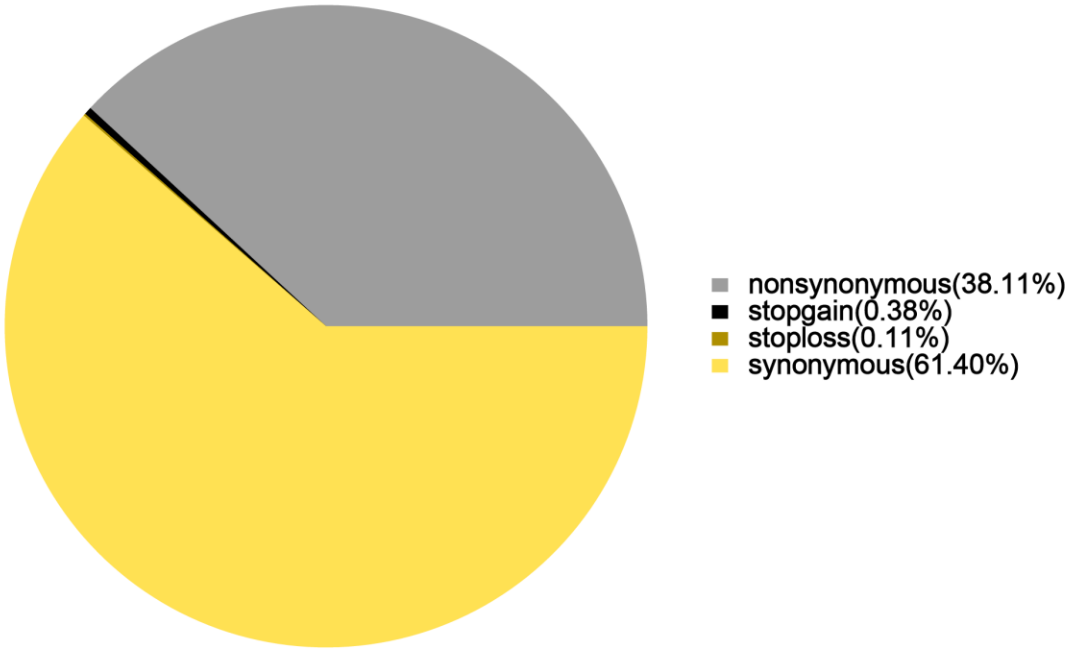

The figures (a) and (b) show the genomic location distribution and functional annotation of the basic SNP set.

Figure S7. The genomic and exonic distribution of the core SNP set.

(a)

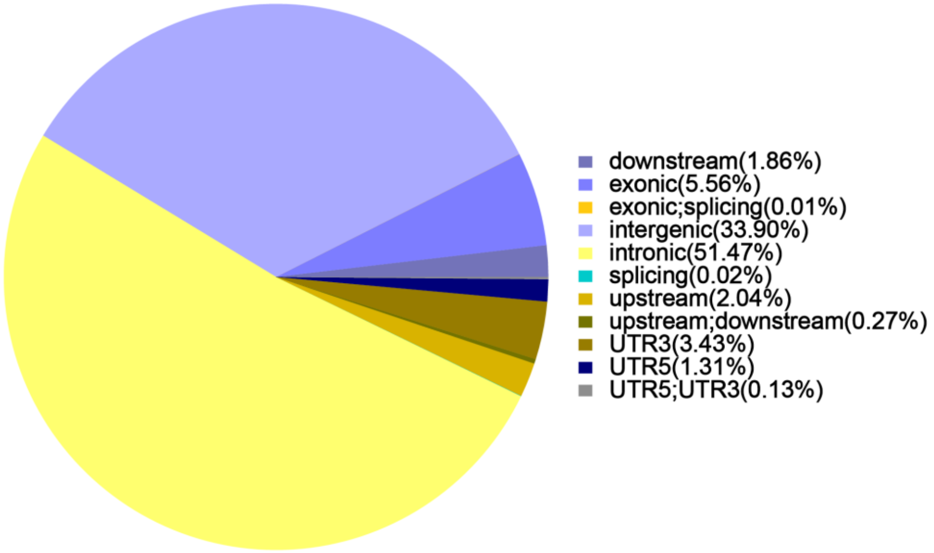

(b)

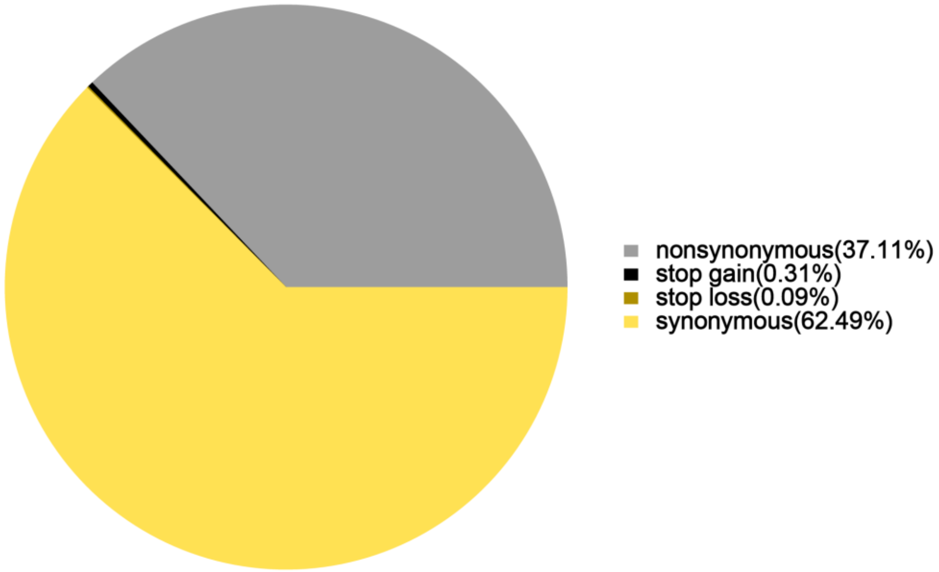

The figures (a) and (b) show the genomic location distribution and functional annotation of the core SNP set.

Figure S8. The genomic distributions of the identified SNPs in each strain.

(a) HB

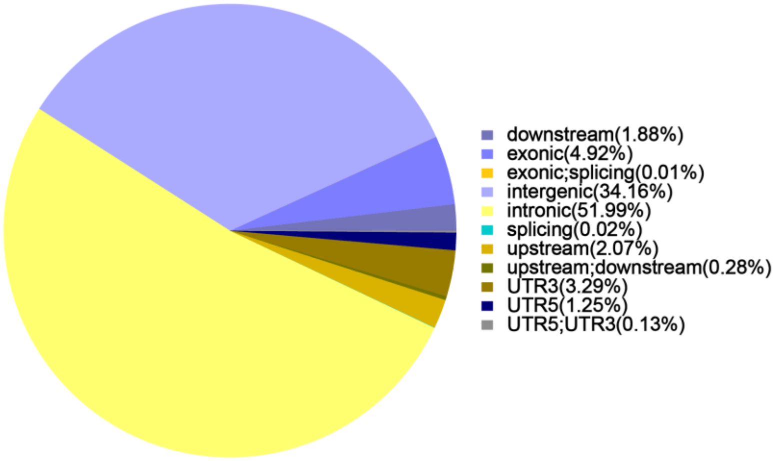

(b) HH

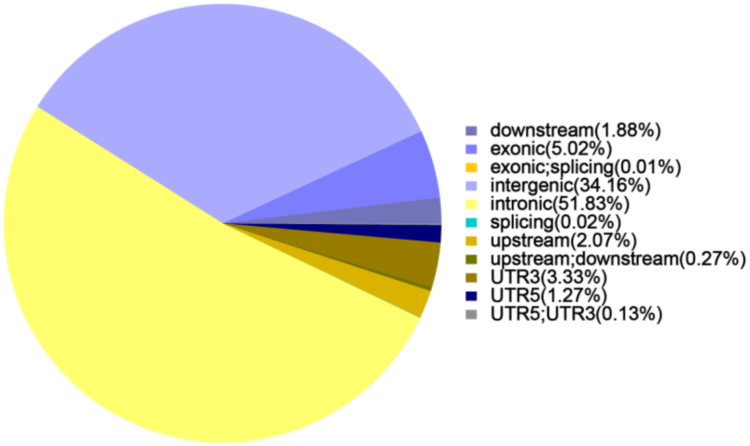

(c) WQ

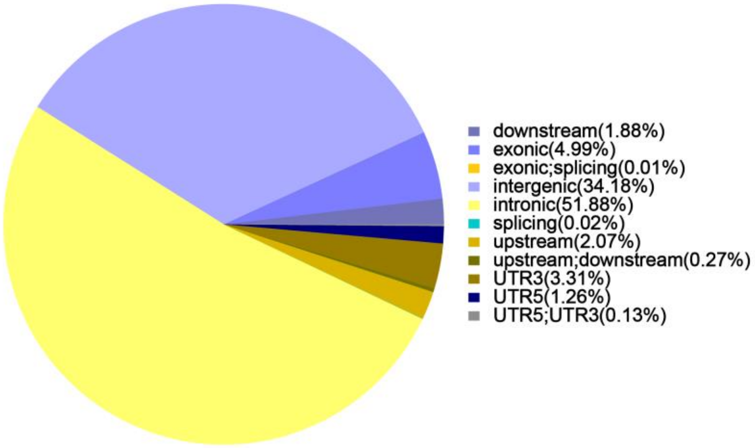

The figure (a), (b), and (c) show the genomic location distributions of SNPs in HB, HH, and WQ strain, respectively.

Figure S9. The function effects of the exonic SNPs in each strain.

(a) HB

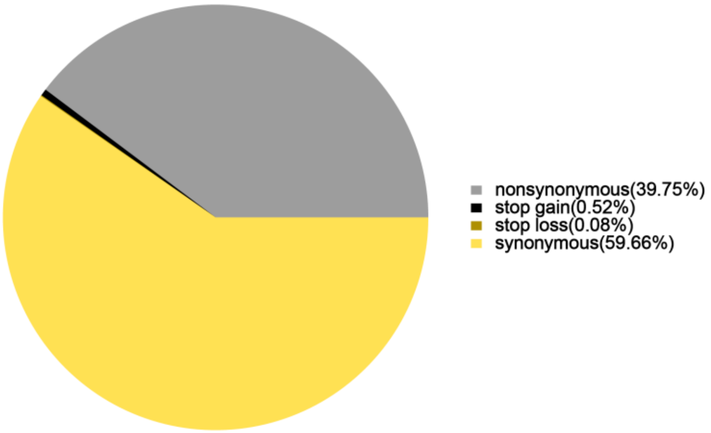

(b) HH

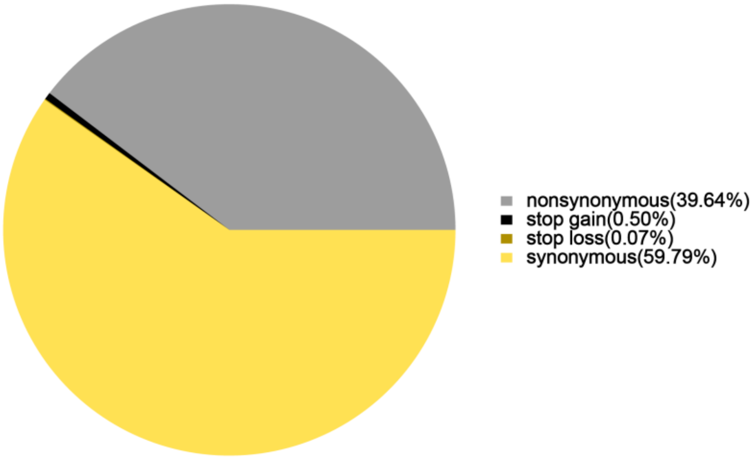

(c) WQ

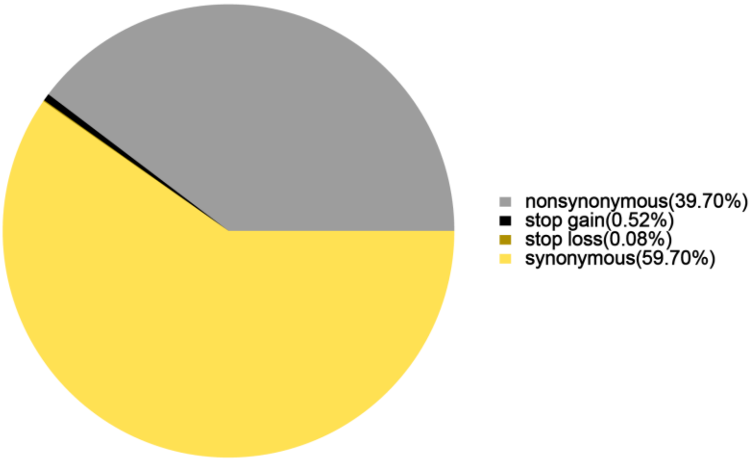

The figures (a), (b), and (c) show the functional annotations of the SNPs in the HB, HH, and WQ strain, respectively.

Figure S10. The genomic distributions of the identified BSA-SNPs in three strains.

(a) HB

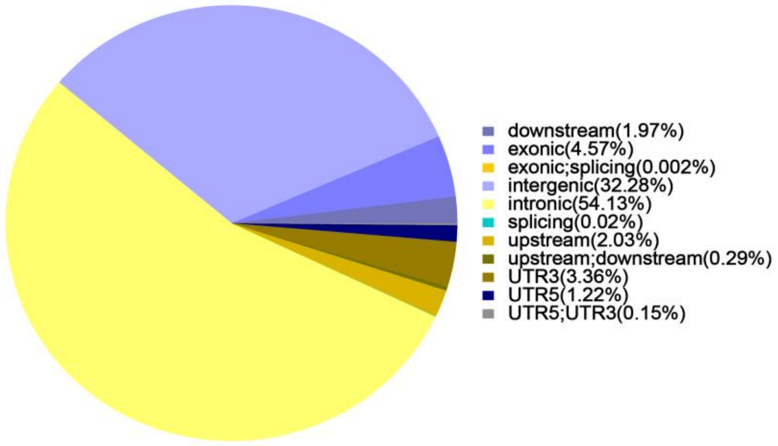

(b) HH

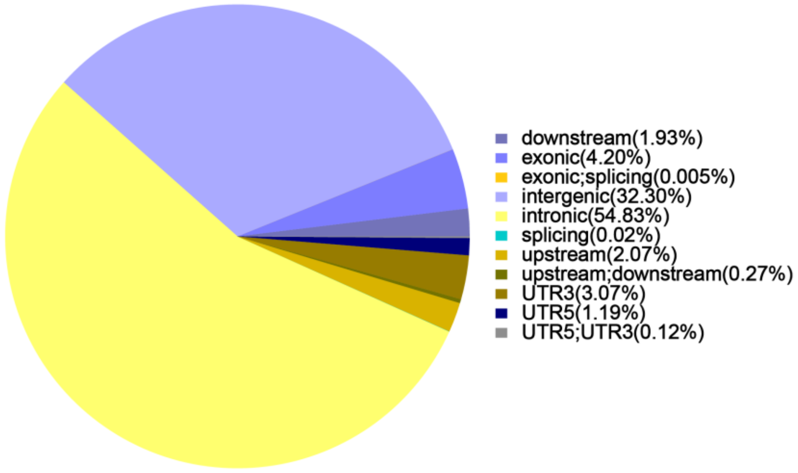

(c) WQ

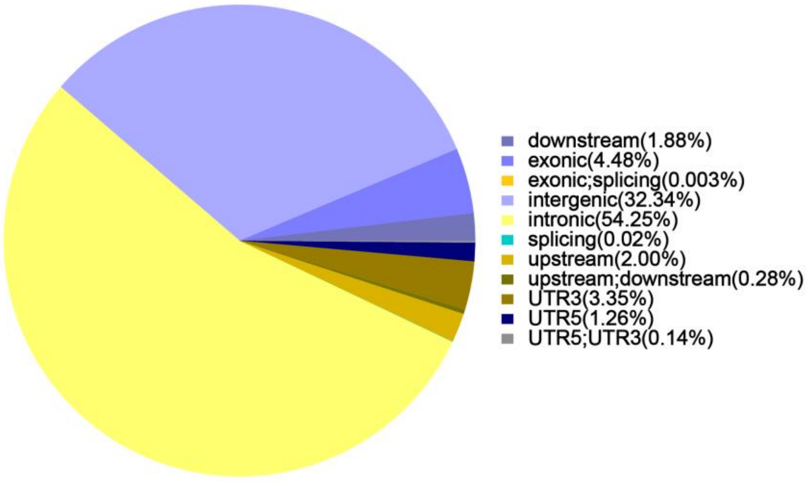

Figure S11. The function effects of the exonic BSA-SNPs in three strains.

(a) HB

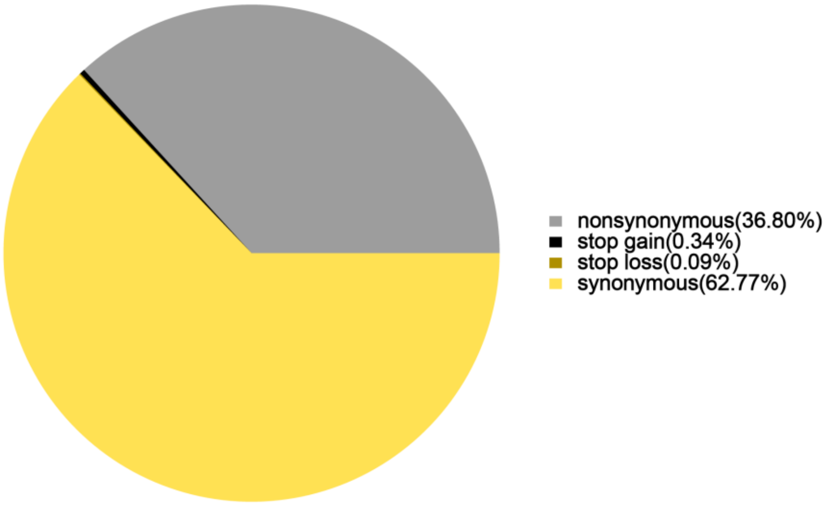

(b) HH

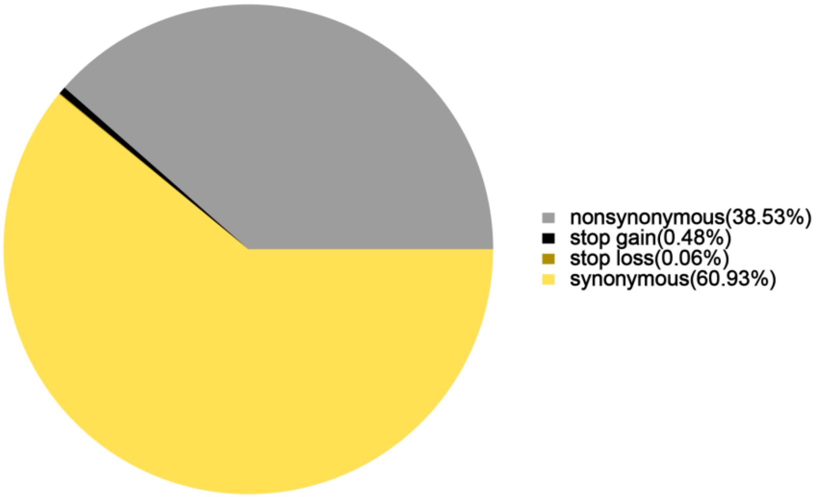

(c) WQ

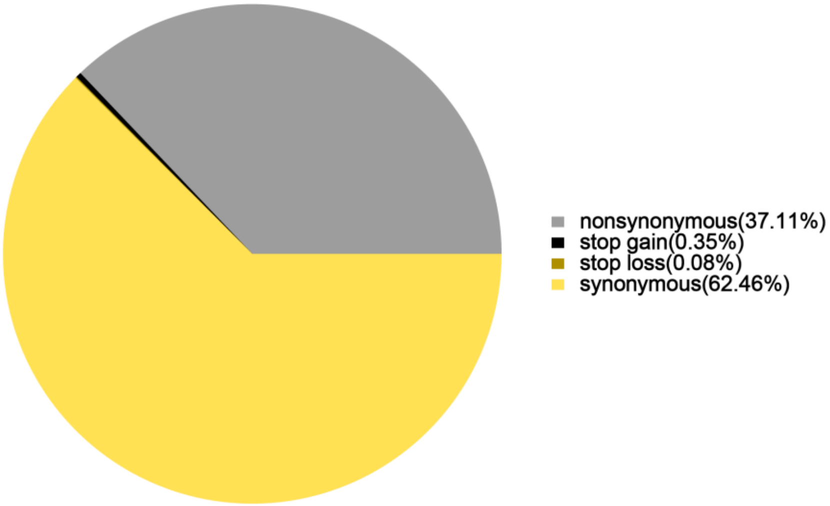

Figure S12. The Venn diagram of the shared BSA-genes in three strains.

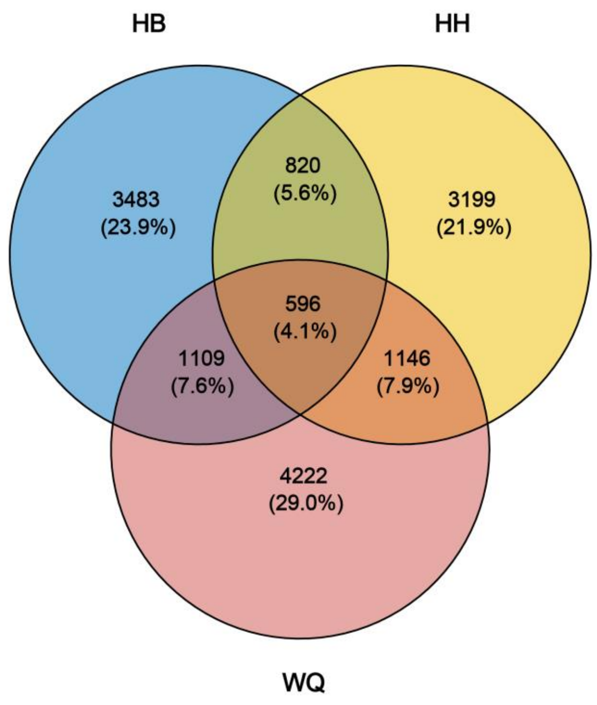

Figure S13. The Venn diagram of the shared GO terms enriched by the genes with the deleterious exonic BSA-SNPs in three strains.

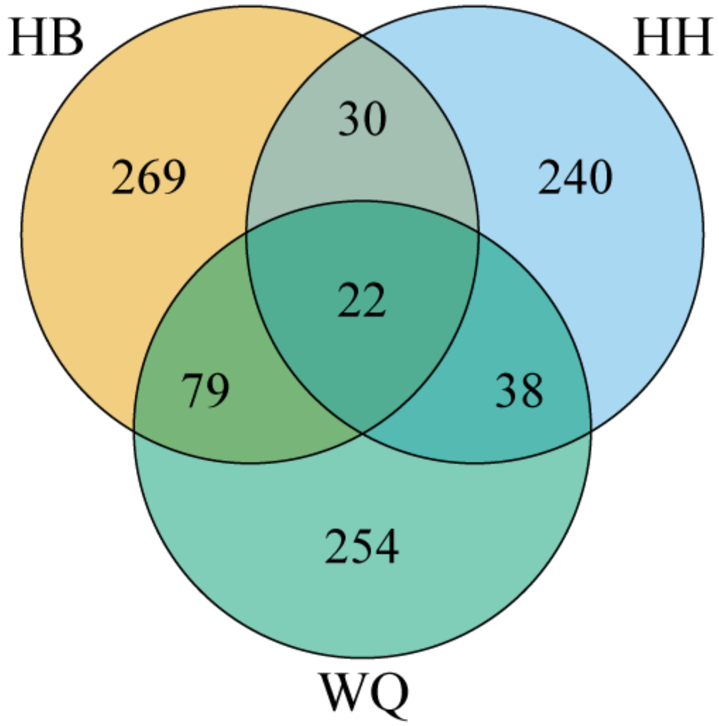

Figure S14. The Venn diagram of the shared BSA-SNPs in three strains.

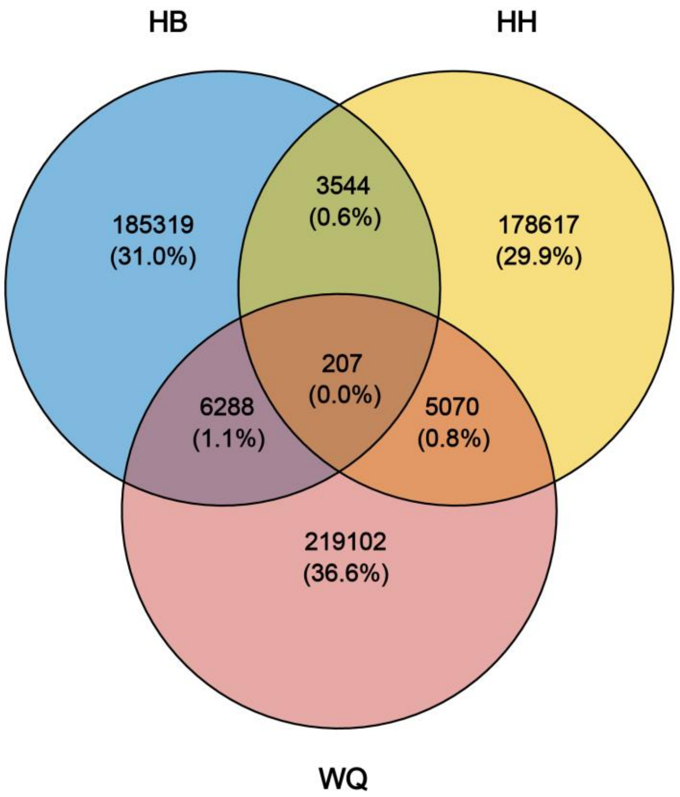

Figure S15. PCA plots detected with the SNPs in three regions, respectively.

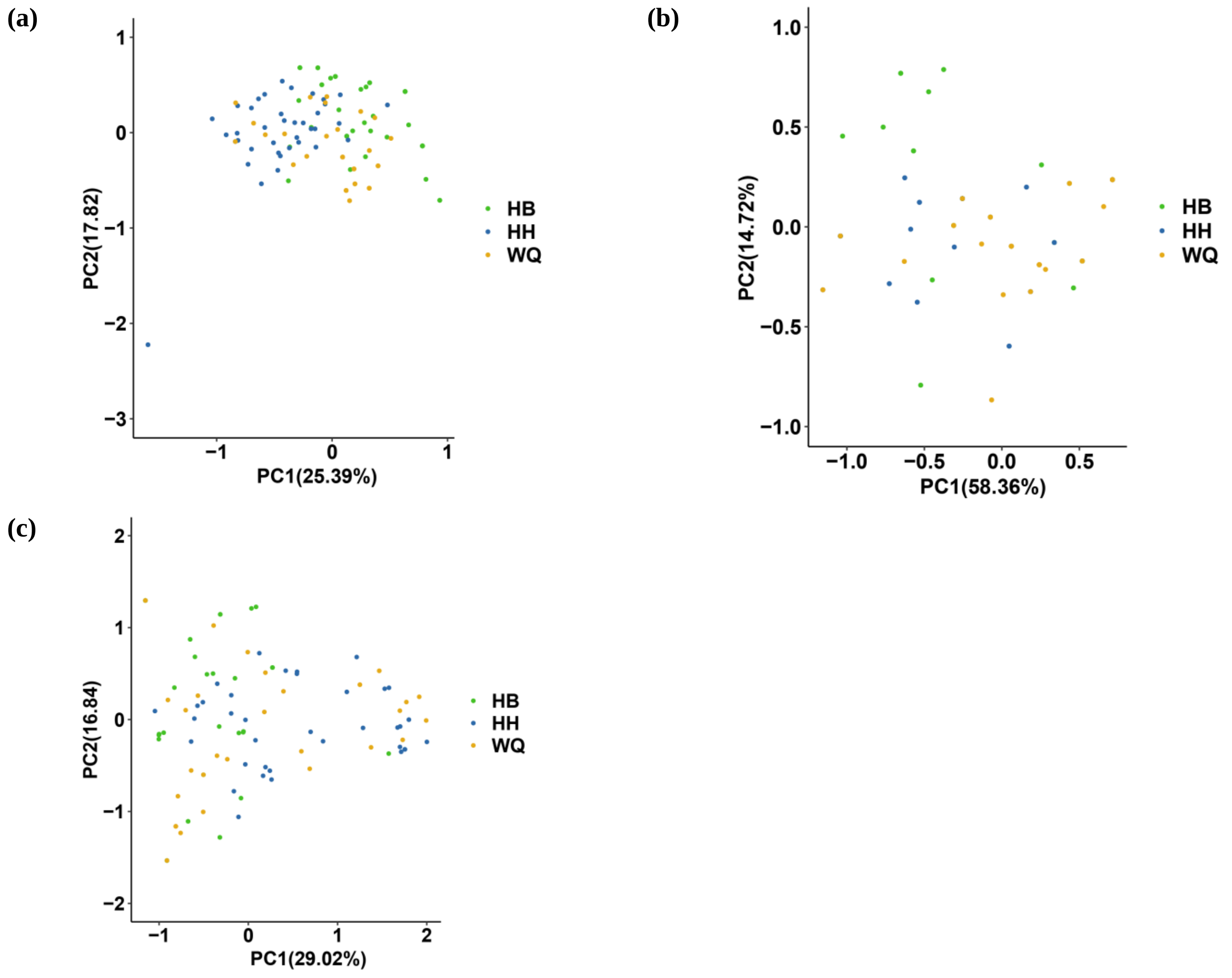

Figure S16. Population structures detected with the SNPs in three regions, respectively.

(a)

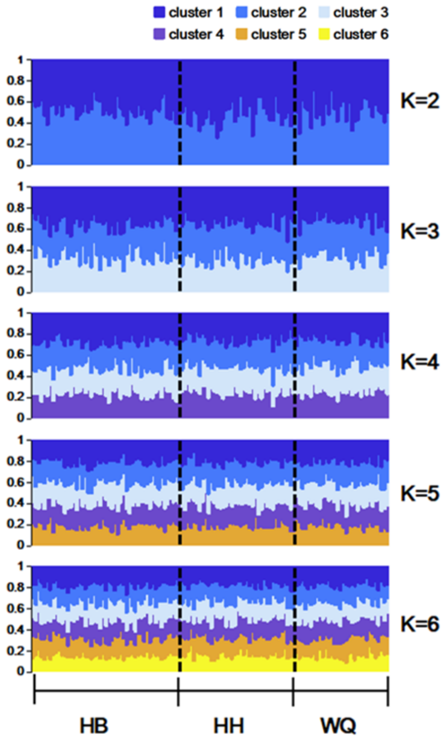

(b)

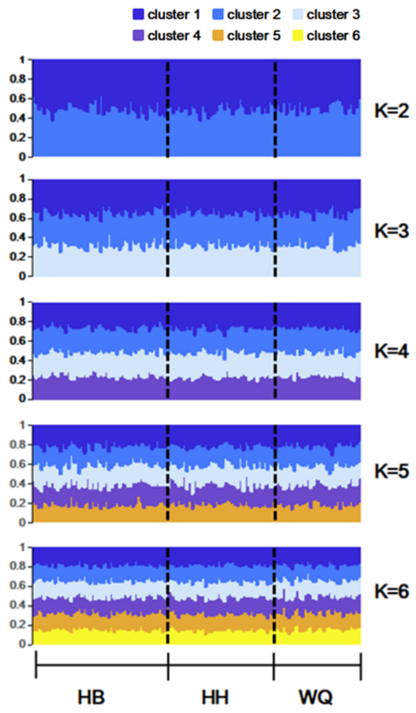

(c)

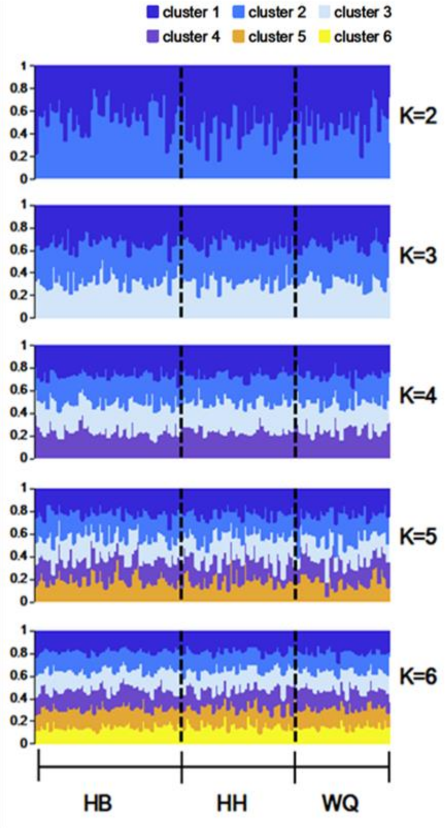

Figure S17. The Manhattan plots showing the SNPs and their associated IB numbers.

(a) two SNPs associated with the number of total IBs, identified with both GLM and ANOVA.

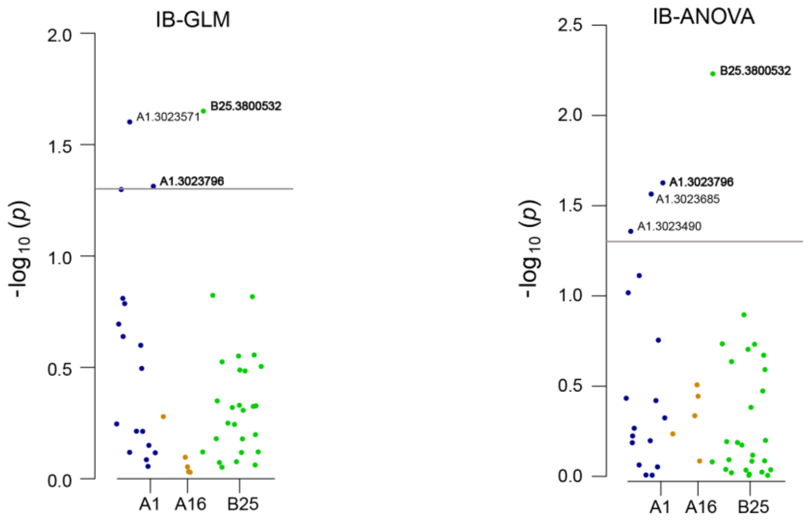

(b) one SNP associated with the number of en-IBs, identified with both GLM and ANOVA.

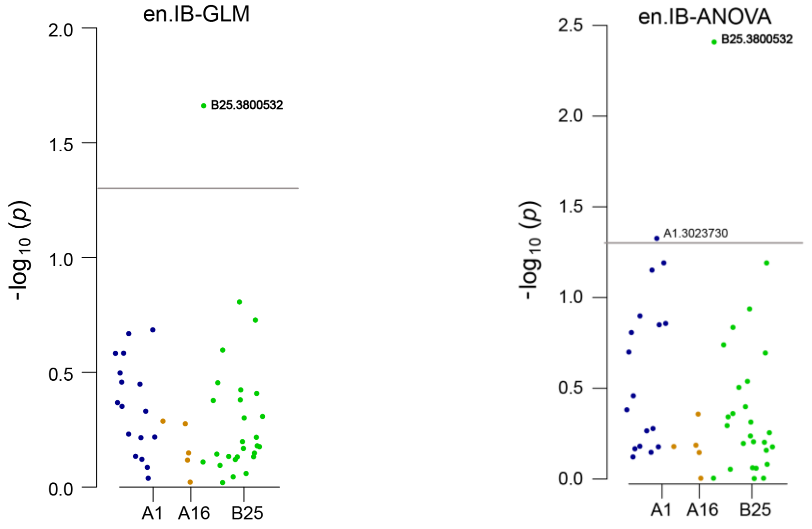

(c) four SNPs associated with the number of en-I IBs, identified with both GLM and ANOVA.

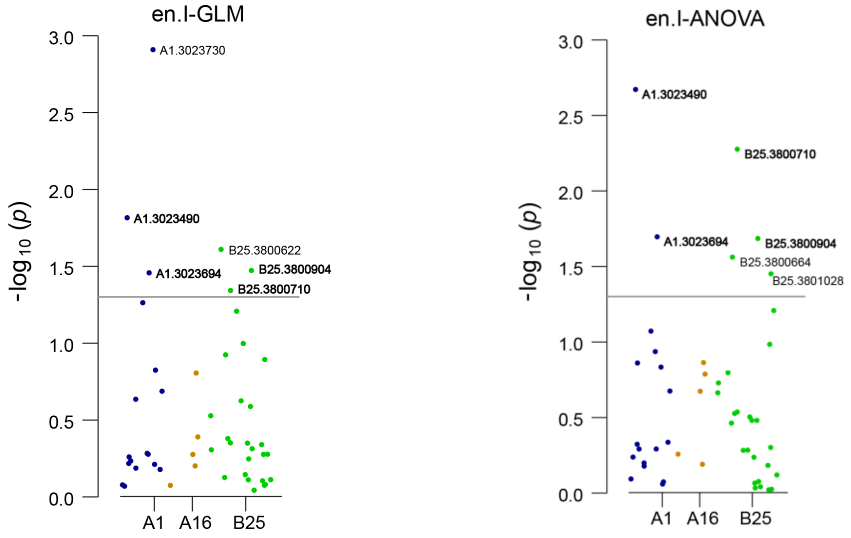

(d) four SNPs associated with the number of en-OEMF IBs, identified with both GLM and ANOVA.

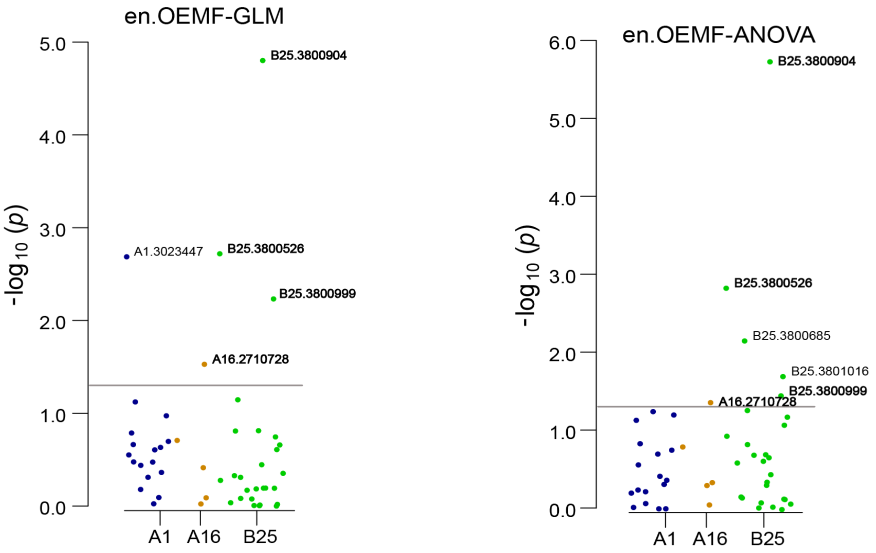

(e) two SNPs associated with the number of en-OEUB IBs, identified with both GLM and ANOVA.

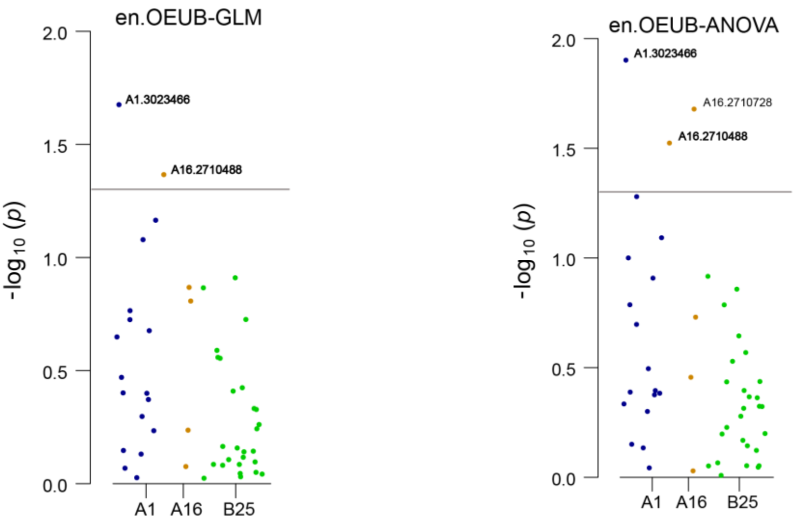

(f) six SNPs associated with the number of en-TEBF IBs, identified with both GLM and ANOVA.

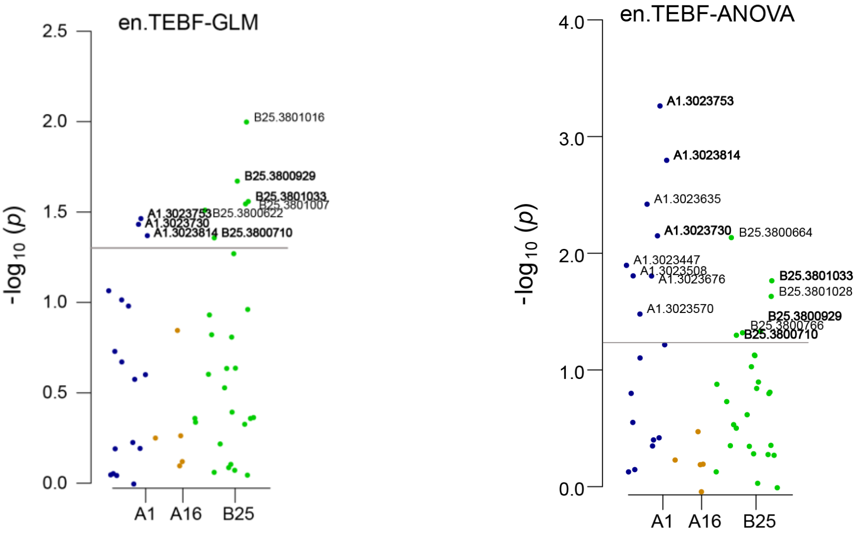

(g) four SNPs associated with the number of en-TEMF IBs, identified with both GLM and ANOVA.

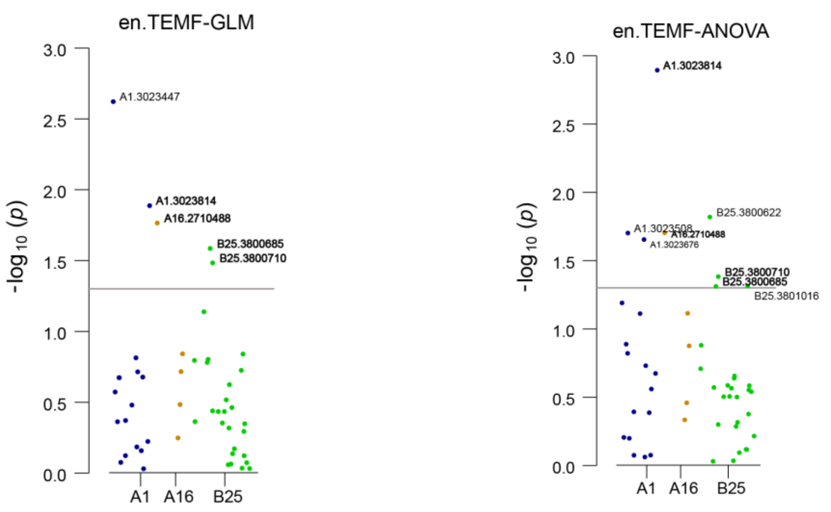

(h) three SNPs associated with the number of en-Y IBs, identified with both GLM and ANOVA.

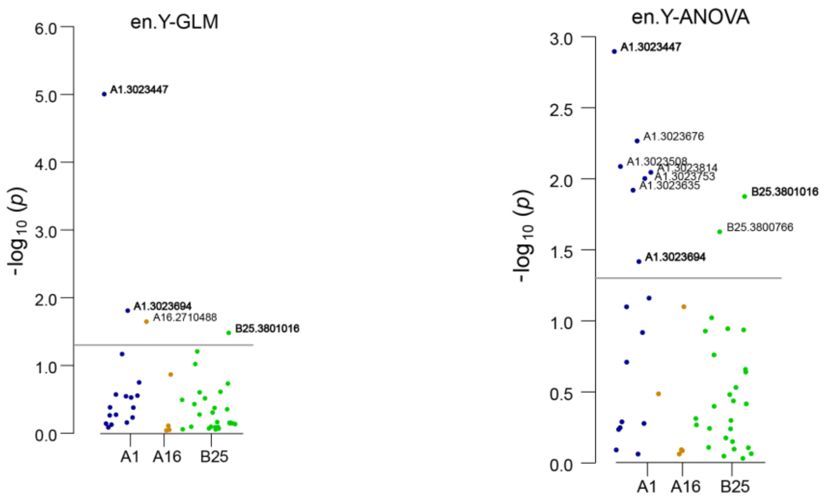

(i) one SNP associated with the number of ep-I IBs, identified with both GLM and ANOVA.

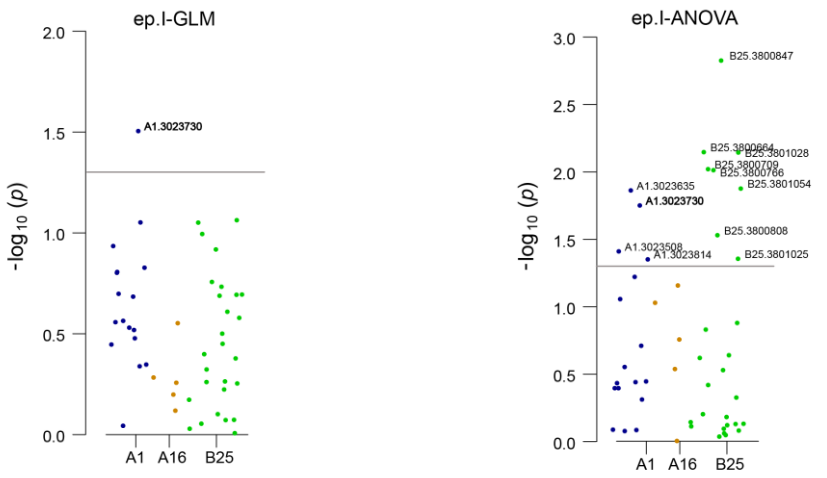

(j) one SNP associated with the number of ep-OEMF IBs, identified with both GLM and ANOVA.

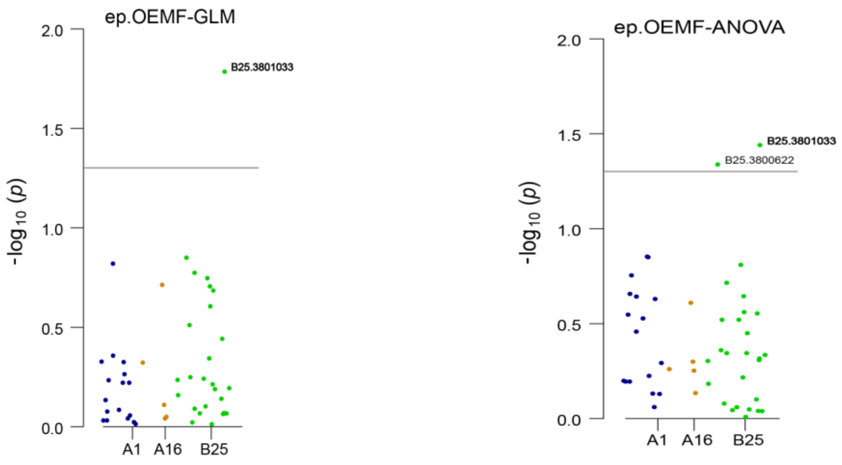

(k) two SNPs associated with the number of ep-TEMF IBs, identified with both GLM and ANOVA.

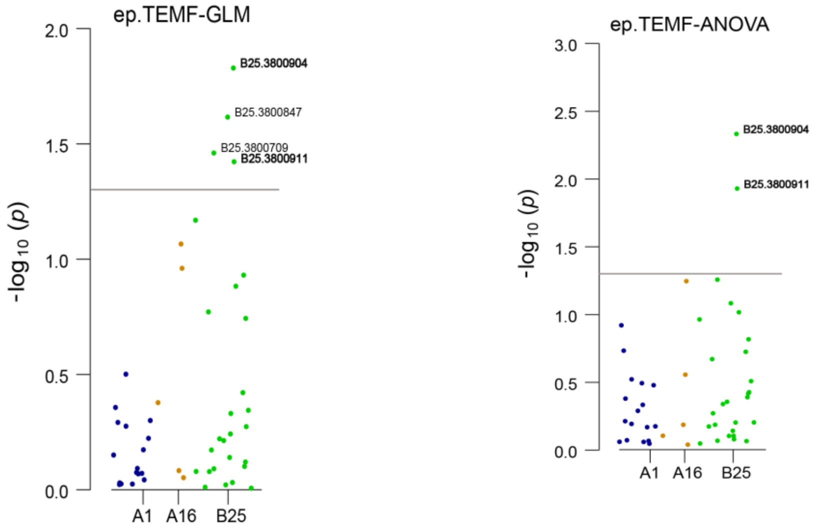

(l) two SNPs associated with the number of ep-Y IBs, identified with both GLM and ANOVA.

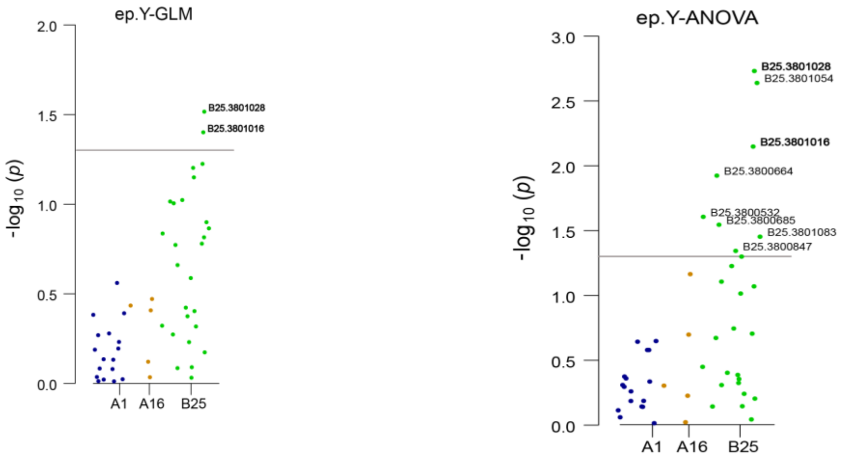

The line represents the p value of 0.05. The SNPs above the line are significantly associated with one IB number. If these SNPs are marked with the bold fonts, they are identified with both GLM and ANOVA methods. The SNPs marked with the normal fonts and located above the line are identified with only one method. The blue, brown, and green dots represent the SNPs located in the A1, A16, and B25 region, respectively. The dots under the line mean that the SNPs are not significantly associated with the IB trait.

Figure S18. The homozygote and heterozygote of each SNP and corresponding IB numbers.

<table>
<tr>
<td>(a)</td>
<td>The homozygote of A16:2710728 was associated with more en-OEMF IBs.
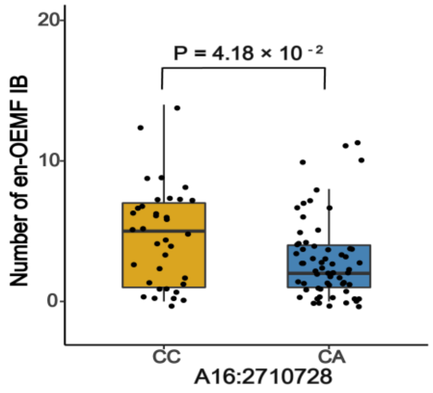
</td>
<td>(b)</td>
<td>The heterozygote of A16:2710488 was associated with more en-OEUB IBs.
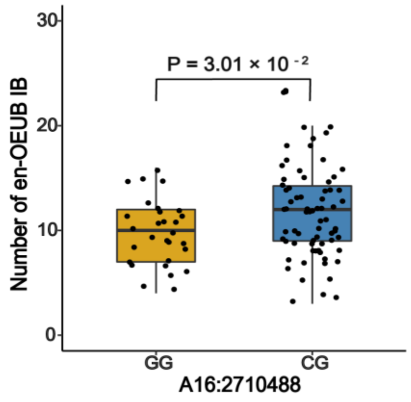
</td>
</tr>
<tr>
<td>(c)</td>
<td>The homozygote of A16:2710488 was associated with more en-TEMF IBs.
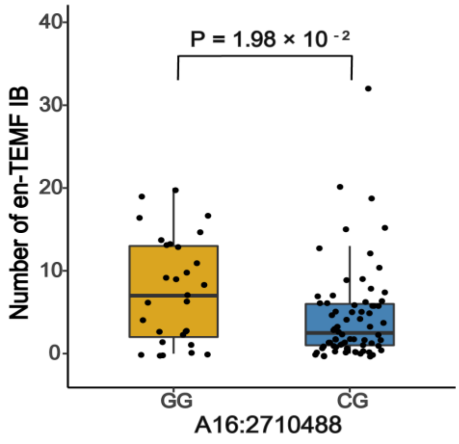
</td>
<td>(d)</td>
<td>The homozygote of A1:3023796 was associated with more total IBs.
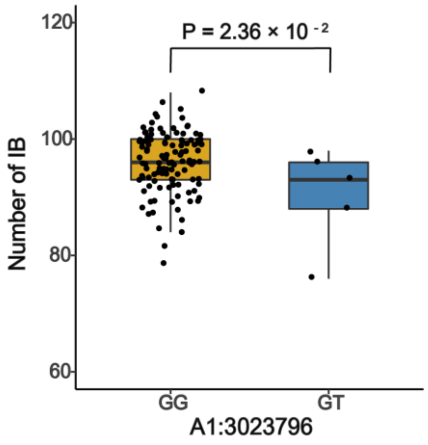
</td>
</tr>
<tr>
<td>(e)</td>
<td>The heterozygote of A1:3023490 was associated with more en-I IBs.
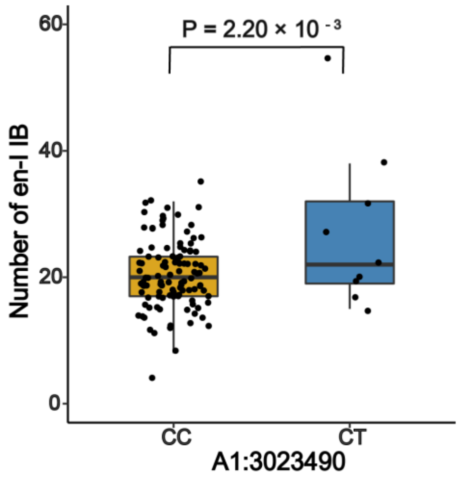
</td>
<td>(f)</td>
<td>The homozygote of A1:3023694 was associated with more en-I IBs.
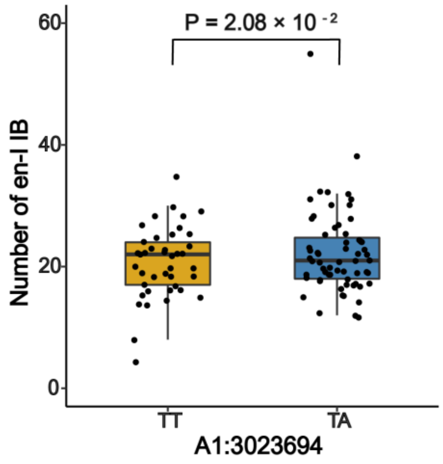
</td>
</tr>
</table>

(g) **The homozygote of A1:3023466 was associated with more en-OEUB IBs.**

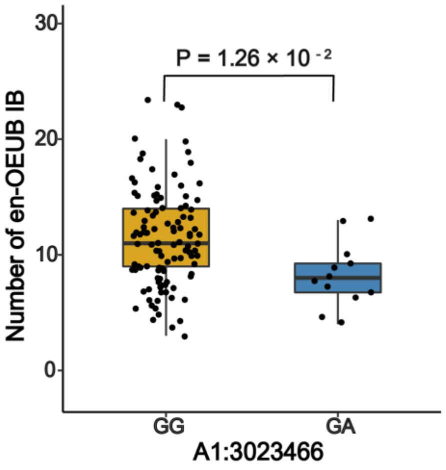

(h) **The homozygote of A1:3023730 was associated with more en-TEBF IBs.**

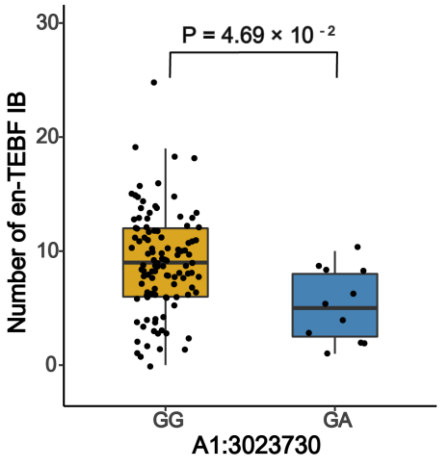

(i) **The homozygote of A1:3023753 was associated with more en-TEBF IBs.**

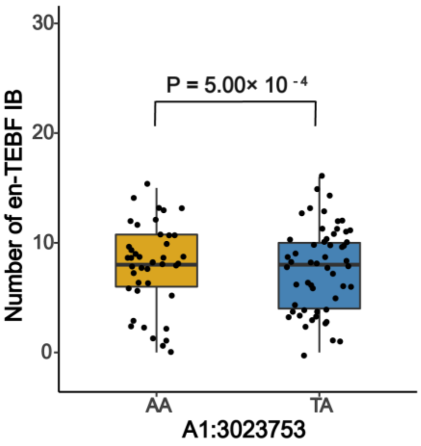

(j) **The homozygote of A1:3023814 was associated with more en-TEBF IBs.**

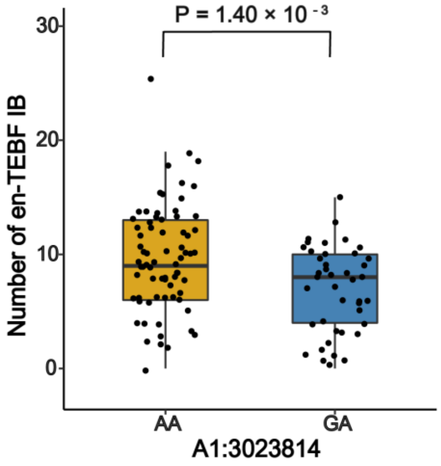

(k) **The homozygote of A1:3023814 was associated with more en-TEMF IBs.**

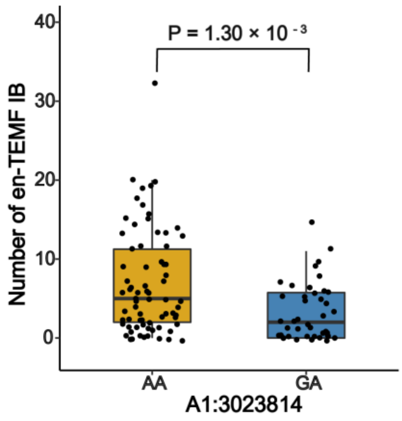

(l) **The heterozygote of A1:3023447 was associated with more en-Y IBs.**

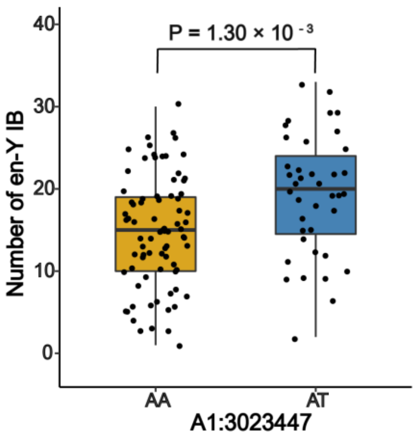

**(m)**	**The heterozygote of A1:3023694 was associated with more en-Y IBs.** 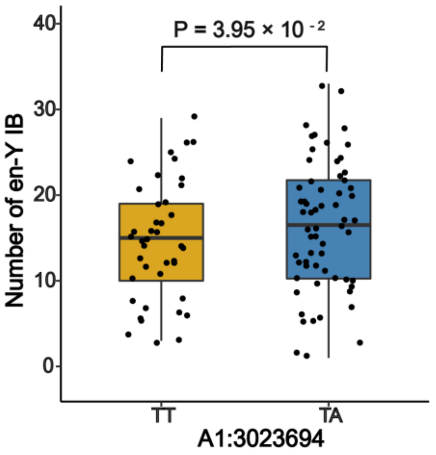	**(n)**	**The heterozygote of A1:3023730 was associated with more ep-I IBs.** 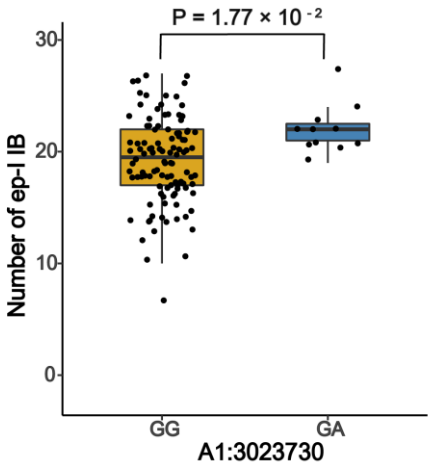
**(o)**	**The homozygote of B25:3800532 was associated with more IBs.** 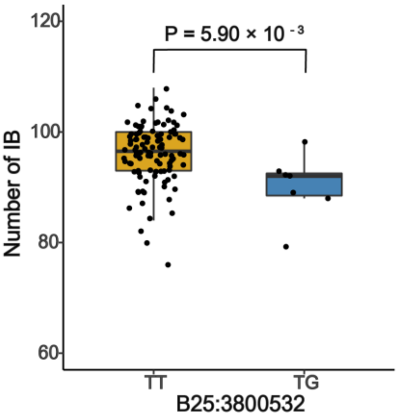	**(p)**	**The homozygote of B25:3800532 was associated with more en-IBs.** 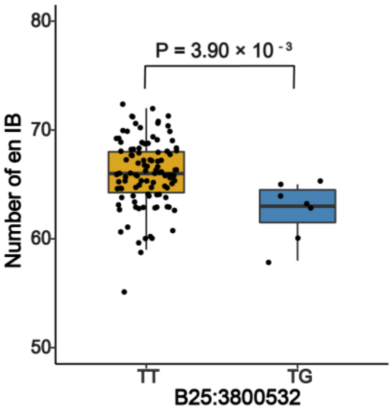
**(q)**	**The homozygote of B25:3800904 was associated with more en-I IBs.** 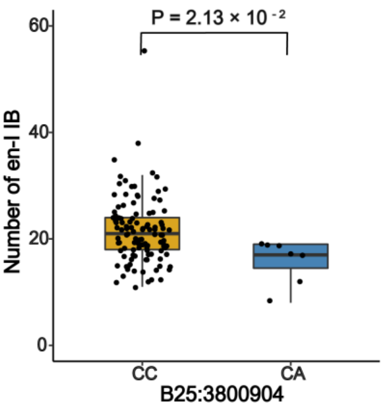	**(r)**	**The heterozygote of B25:3800710 was associated with more en-I IBs.** 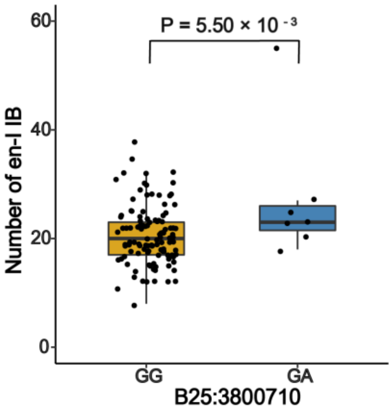

(s) The heterozygote of B25:3800526 was associated with more en-OEMF IBs.

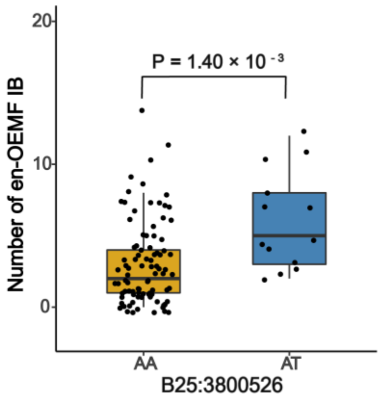

(t) The heterozygote of B25:3800904 was associated with more en-OEMF IBs.

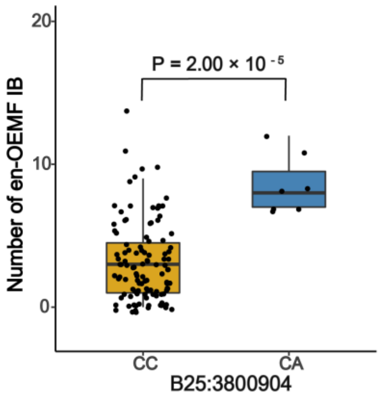

(u) The heterozygote of B25:3800999 was associated with more en-OEMF IBs.

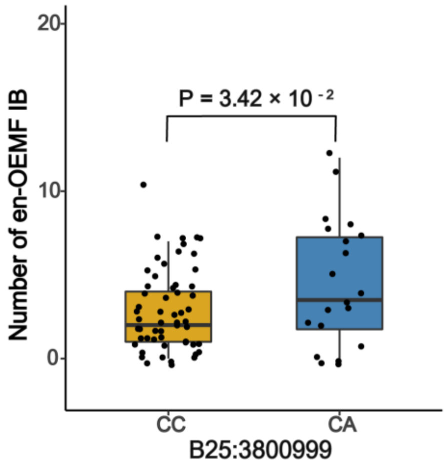

(v) The homozygote of B25:3800710 was associated with more en-TEBF IBs.

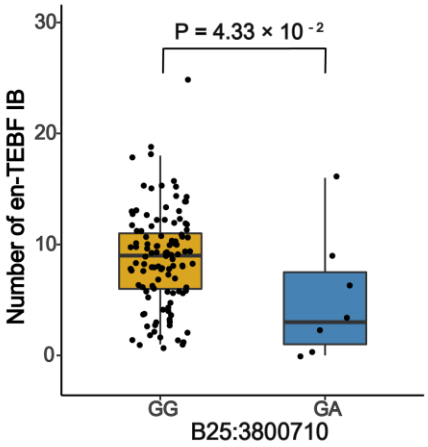

(w) The heterozygote of B25:3800929 was associated with more en-TEBF IBs.

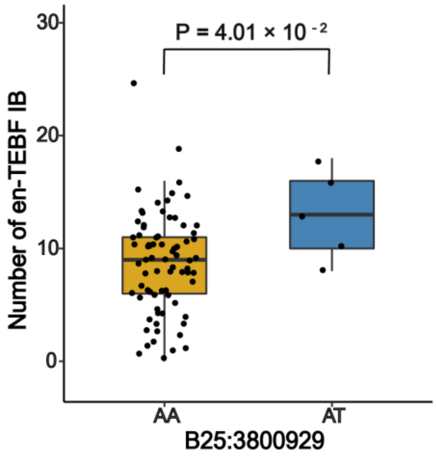

(x) The heterozygote of B25:3801033 was associated with more en-TEBF IBs.

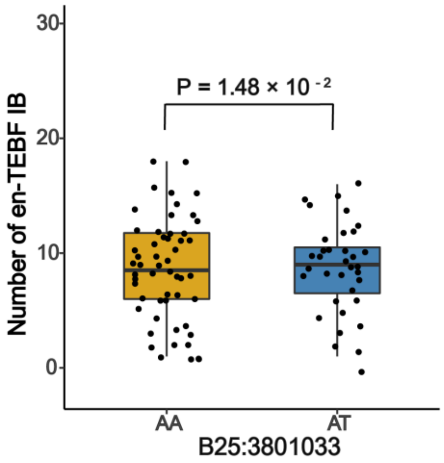

(y)	The homozygote of B25:3800685 was associated with more en-TEMF IBs. 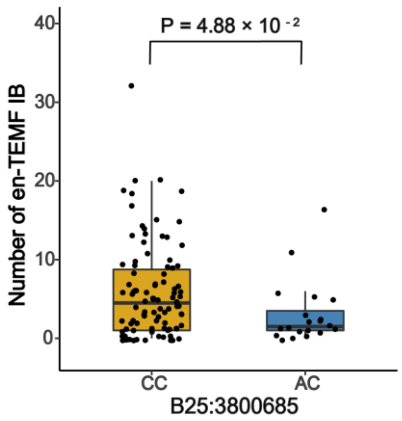	(z)	The homozygote of B25:3800710 was associated with more en-TEMF IBs. 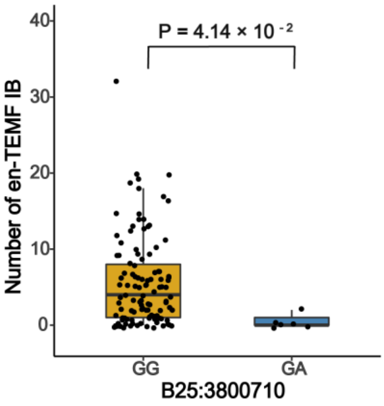
(aa)	The heterozygote of B25:3801016 was associated with more en-Y IBs. 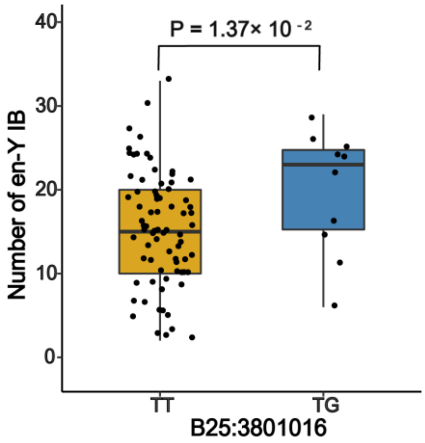	(ab)	The heterozygote of B25:3801033 was associated with more ep-OEMF IBs. 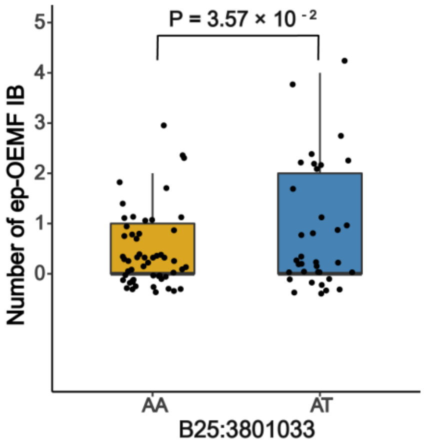
(ac)	The heterozygote of B25:3800904 was associated with more ep-TEMF IBs. 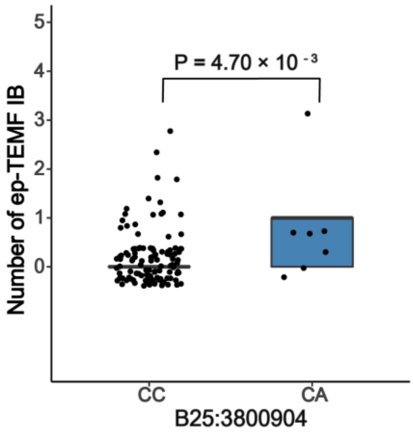	(ad)	The heterozygote of B25:3800911 was associated with more ep-TEMF IBs. 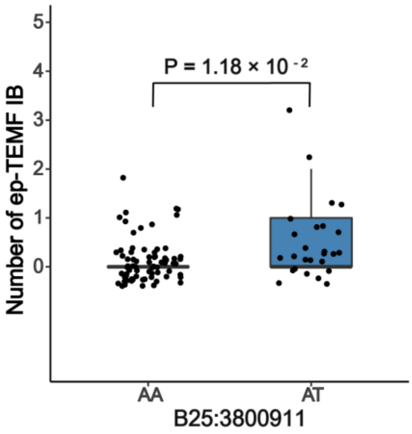

**(ae)**	**The homozygote of B25:3801016 was associated with more ep-Y IBs.** 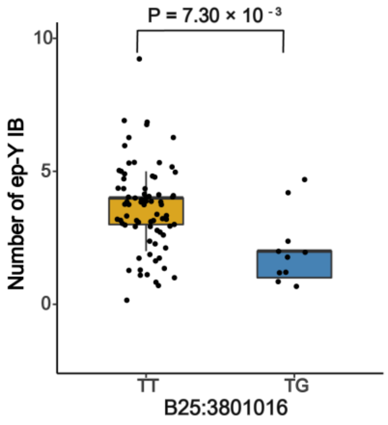	**(af)**	**The homozygote of B25:3801028 was associated with more ep-Y IBs.** 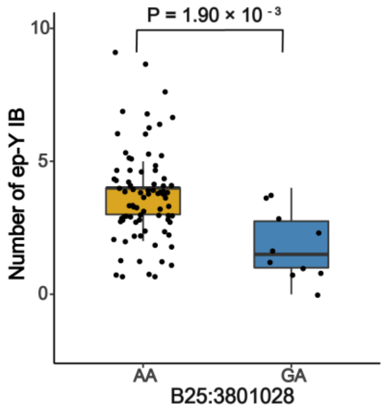

Figure S19. The genotype combinations and corresponding IB numbers.

(a) **Three genotype combinations in two SNP loci and corresponding numbers of total IBs.**

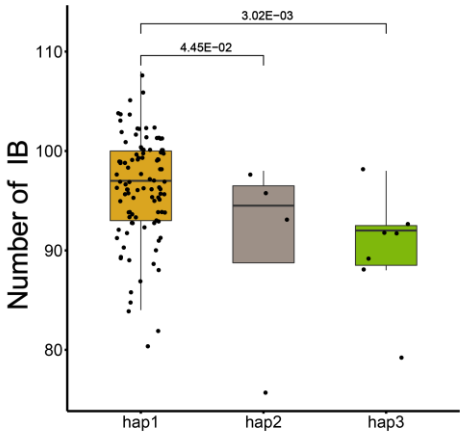

(b) **Seven genotype combinations in four SNP loci and corresponding numbers of en-I IBs.**

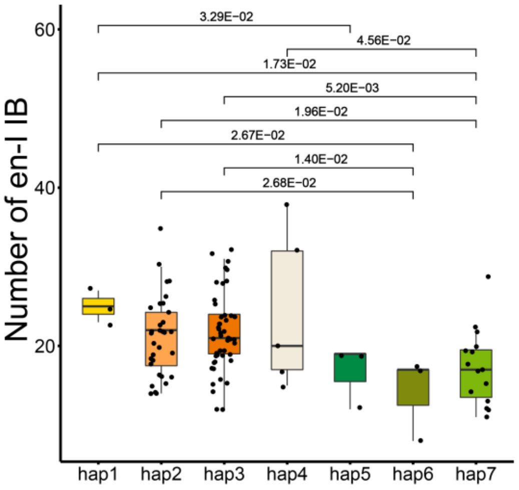

(c) **Five genotype combinations in four SNP loci and corresponding numbers of en-OEMF IBs.**

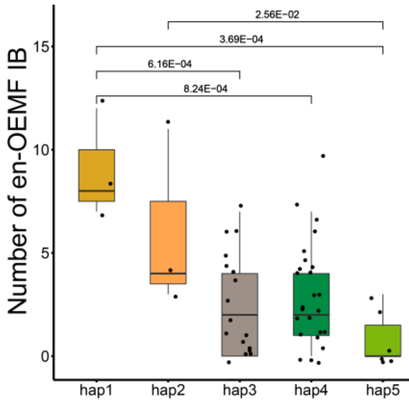

(d) **Four genotype combinations in two SNP loci and corresponding numbers of en-OEUB IBs.**

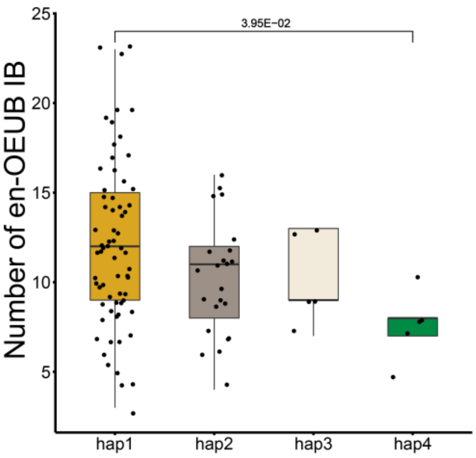

(e) **Twelve genotype combinations in six SNP loci and corresponding numbers of en-TEBF IBs.**

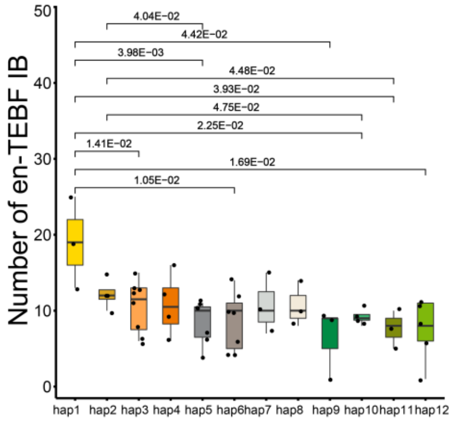

(f) **Eight genotype combinations in four SNP loci and corresponding numbers of en-TEMF IBs.**

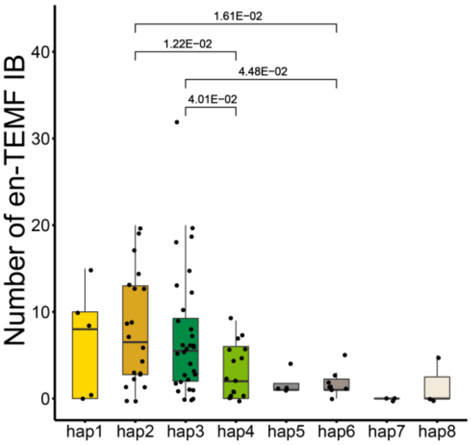

(g) Six genotype combinations in three SNP loci and corresponding numbers of en-Y IBs.

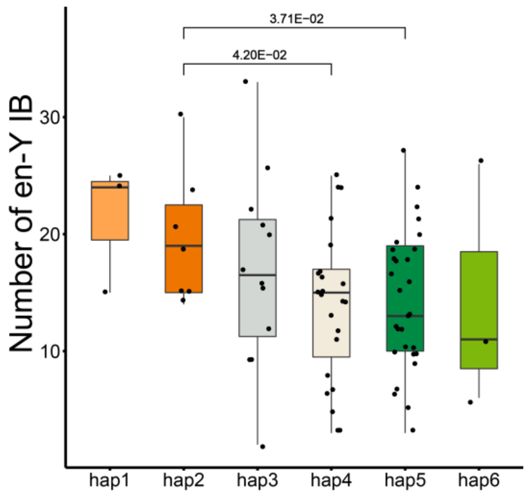

(h) Three genotype combinations in two SNP loci and corresponding numbers of ep-TEMF IBs.

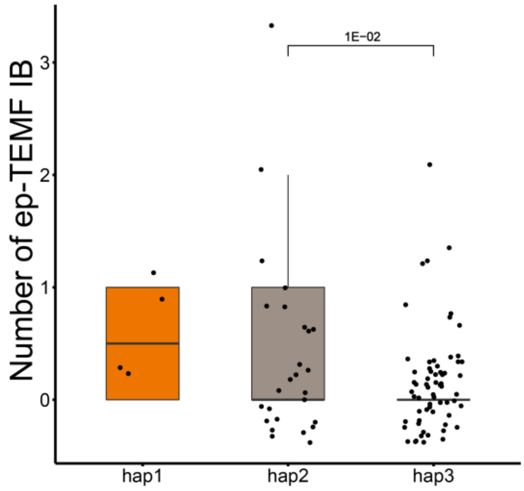

(i) Four genotype combinations in two SNP loci and corresponding numbers of ep-Y IBs.

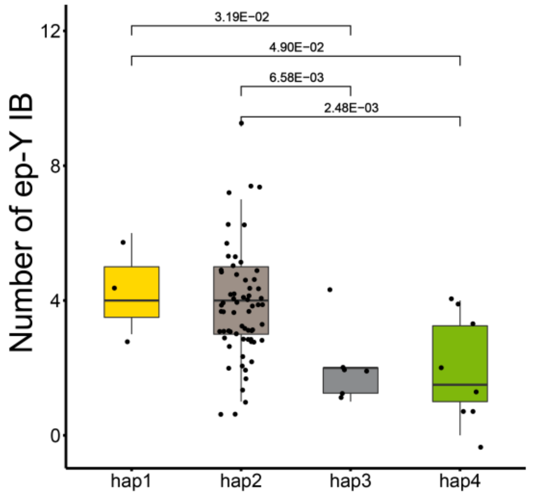

Figure S20. The BMP-related genes identified with BSA in each strain.

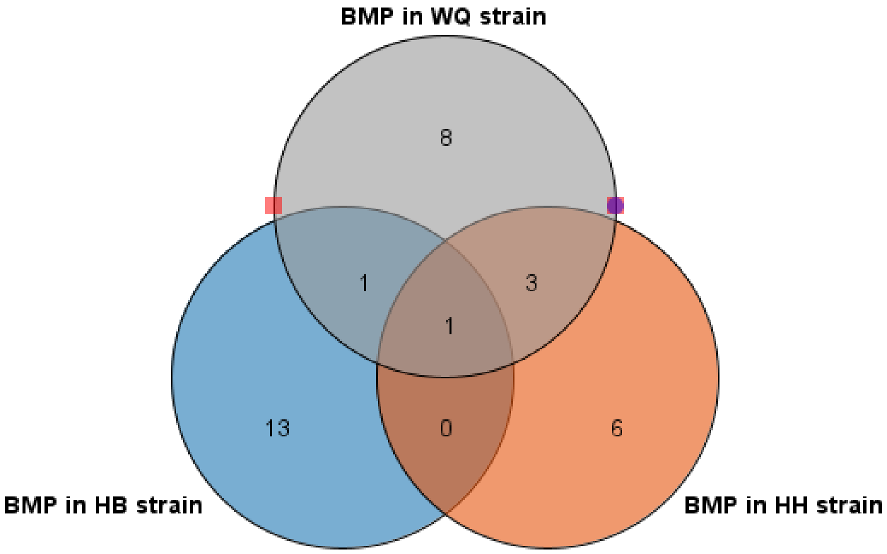

Supplementary Tables

Table S1. The primers used for amplification of the BSA-SNP regions.

Locus	Primer sequence (5'-3')	Tm	Amplification length (bp)
A1:3023753	F:GTTCAGACTGCTGCTTGCTTC	55	516
	R: CTATGAGCTCACCGCCTCTT		
A16:2710463	F:ACCCTAAGGCATAGCTCAACG	55	564
	R: ACTCCTGGATGCACTTGTGT		
B25:3800828	F: TGTAGCTCCTCGAGGTTGGTT	55	683
	R: ATCAGATCTCTGTGTGGAGCAC		

Table S2. The IB numbers in 122 common carp individuals from three strains (in a separated excel file).

Table S3. The identified BSA-SNPs in each strain.

Strain	IB-fewer group	IB-more group	Total	Overlapping SNPs	Significant BSA-SNPs	Overlapping significant BSA-SNPs
HH	9,225,546	9,321,881	12,531,928	4,812,113	195,357	207
HB	8,511,519	8,611,217	10,835,374		187,438	
WQ	8,763,283	8,644,975	11,477,507		230,667	

Table S4. Slight higher Ts/Tv ratio and C/T transition in the BSA-SNPs than all SNPs in each strain.

	**Ts/Tv in all SNPs**	**Ts/Tv in the BSA-SNPs**	**C/T transition in all SNPs**	**C/T transition in BSA-SNP**
**HB**	2.222	2.351	34.5%	35%
**HH**	2.254	2.337	34.6%	35.2%
**WQ**	2.241	2.316	34.6%	35.1%

Table S5. The enriched GO terms by the genes with the deleterious exonic BSA-SNPs in three strains.

Table S6. The functional annotations of 318 genes neighboring the shared 207 BSA-SNPs in three strains (in a separated excel file).

Table S7. The genotypes of three loci in 122 common carp individuals from three strains (in a separated excel file).

Table S8. Genetic diversities of three sequenced regions.

**SNP locus**	**Ref/Alt**	**Ho**	**He**	**PIC**	**MAF**	**Genotype**	**Frequencies of genotypes**	**Number**	**Classification**
A1.3023447	A/T	0.32	0.29	0.25	0.18	AA	0.661	80	Intronic
						AT	0.322	39	
A1.3023466	G/A	0.1	0.09	0.09	0.05	GA	0.099	12	Syn, A->A

						GG	0.901	109	
A1.3023490	C/T	0.07	0.07	0.07	0.04	CC	0.926	112	Syn, D->D
						CT	0.074	9	
A1.3023505	C/T	0.1	0.09	0.09	0.05	CC	0.901	109	Syn, S->S
						CT	0.099	12	
A1.3023508	C/T	0.12	0.12	0.11	0.06	CC	0.876	106	Non-syn, P->L
						CT	0.124	15	
A1.3023523	C/T	0.04	0.06	0.05	0.03	CC	0.95	115	Syn, H->H
						CT	0.041	5	
A1.3023570	G/A	0.28	0.28	0.24	0.17	AA	0.694	84	Intronic
						GA	0.281	34	
A1.3023571	C/G/T	0.34	0.34	0.3	0.03	CC	0.628	76	Intronic
						CG	0.273	33	
						GG	0.033	4	
						CT	0.066	8	
A1.3023635	C/T	0.15	0.15	0.14	0.08	CC	0.843	102	Intronic
						CT	0.149	18	
A1.3023676	G/A	0.14	0.13	0.12	0.07	GA	0.14	17	Syn, K->K
						GG	0.86	104	
A1.3023685	T/C	0.31	0.3	0.26	0.19	CC	0.661	80	Syn, P->P
						TC	0.306	37	
						TT	0.033	4	
A1.3023694	T/A	0.51	0.48	0.37	0.4	AA	0.149	18	Syn, T->T
						TA	0.512	62	
						TT	0.339	41	
A1.3023730	G/A	0.09	0.09	0.08	0.05	GA	0.092	11	Syn, R->R
						GG	0.908	108	
A1.3023745*	G/A	0.29	0.27	0.23	0.16	GA	0.289	35	Non-Syn, M->I
						GG	0.694	84	
A1.3023753*	T/A	0.45	0.49	0.37	0.43	AA	0.347	42	Non-Syn, L->Q
						TA	0.446	54	
						TT	0.207	25	
A1.3023796	G/T	0.04	0.04	0.04	0.02	GG	0.959	116	Syn, V->V
						GT	0.041	5	
A1.3023814	G/A	0.35	0.36	0.29	0.23	AA	0.595	72	Syn, A->A
						GA	0.347	42	
						GG	0.058	7	
B25.3800526	A/T	0.12	0.2	0.18	0.11	AA	0.826	90	Intronic
						AT	0.119	13	
						TT	0.055	6	
B25.3800532	T/G	0.06	0.06	0.06	0.03	TG	0.062	7	Intronic
						TT	0.938	106	

B25.3800622	G/C	0.27	0.29	0.25	0.18	CC	0.042	5	Syn, S->S
						GC	0.267	32	
						GG	0.692	83	
B25.3800655	C/T	0.33	0.44	0.35	0.33	CC	0.5	60	Syn, C->C
						CT	0.333	40	
						TT	0.167	20	
B25.3800664	C/G	0.26	0.31	0.27	0.2	CC	0.675	81	Syn, T->T
						CG	0.258	31	
						GG	0.067	8	
B25.3800685	A/C	0.17	0.18	0.16	0.1	AC	0.167	20	Syn, T->T
						CC	0.817	98	
B25.3800709	C/T	0.27	0.34	0.28	0.22	CC	0.647	77	Syn, P->P
						CT	0.269	32	
						TT	0.084	10	
B25.3800710*	G/A	0.06	0.07	0.07	0.04	GA	0.059	7	Non-Syn, V->M
						GG	0.933	111	
B25.3800766	C/T	0.16	0.2	0.18	0.11	CC	0.808	97	Syn, N->N
						CT	0.158	19	
						TT	0.033	4	
B25.3800808	C/T	0.4	0.45	0.35	0.33	CC	0.463	56	Syn, D->D
						CT	0.405	49	
						TT	0.132	16	
B25.3800828*	C/G	0.24	0.31	0.26	0.19	CC	0.074	9	Non-Syn, T->S
						CG	0.24	29	
						GG	0.686	83	
B25.3800847	A/C	0.25	0.33	0.27	0.21	AA	0.669	81	Syn, L->L
						AC	0.248	30	
						CC	0.083	10	
B25.3800867	A/G	0.05	0.06	0.06	0.03	AA	0.942	114	Intronic
						AG	0.05	6	
B25.3800875	A/G	0.37	0.41	0.33	0.29	AA	0.521	63	Intronic
						AG	0.372	45	
						GG	0.107	13	
B25.3800879	C/T	0.38	0.41	0.33	0.29	CC	0.517	62	Intronic
						CT	0.383	46	
						TT	0.1	12	
B25.3800895	A/G	0.4	0.47	0.36	0.37	AA	0.43	52	Intronic
						AG	0.405	49	
						GG	0.165	20	
B25.3800904	C/A	0.06	0.1	0.1	0.05	CA	0.058	7	Intronic
						CC	0.917	111	
B25.3800911	A/T	0.27	0.24	0.21	0.14	AA	0.724	71	Intronic

						AT	0.265	26	
B25.3800929	A/T	0.06	0.08	0.08	0.04	AA	0.927	76	Intronic
						AT	0.061	5	
B25.3800999	C/A	0.24	0.35	0.29	0.22	AA	0.106	9	Intronic
						CA	0.235	20	
						CC	0.659	56	
B25.3801007	G/T	0.36	0.49	0.37	0.43	GG	0.391	34	Intronic
						GT	0.356	31	
						TT	0.253	22	
B25.3801016	T/G	0.11	0.18	0.16	0.1	GG	0.044	4	Intronic
						TG	0.11	10	
						TT	0.846	77	
B25.3801025	T/G	0.04	0.06	0.06	0.03	TG	0.041	4	Intronic
						TT	0.948	92	
B25.3801028	G/A	0.11	0.16	0.14	0.09	AA	0.862	81	Intronic
						GA	0.106	10	
B25.3801033	A/T	0.37	0.37	0.3	0.25	AA	0.568	54	Intronic
						AT	0.368	35	
						TT	0.063	6	
B25.3801054	A/T	0.3	0.38	0.3	0.25	AA	0.098	9	Intronic
						AT	0.304	28	
						TT	0.598	55	
B25.3801083	T/G	0.37	0.48	0.36	0.4	GG	0.419	39	Intronic
						TG	0.366	34	
						TT	0.215	20	
A16.2710488	C/G	0.69	0.47	0.36	0.38	CG	0.692	72	Syn, L->L
						GG	0.279	29	
A16.2710697	A/G	0.3	0.27	0.23	0.16	AG	0.304	31	Intronic
						GG	0.686	70	
A16.2710718	A/G	0.31	0.29	0.25	0.17	AA	0.67	67	Intronic
						AG	0.31	31	
A16.2710728	C/A	0.61	0.45	0.35	0.34	AA	0.039	4	Intronic
						CA	0.612	63	
						CC	0.35	36	
A16.2710743	T/C	0.11	0.1	0.1	0.06	TC	0.111	13	Intronic
						TT	0.889	104	

The locus with the asterisk means that it is non-synonymous.

Table S9. The joint effects of the associated SNPs.

Trait	Hap name	Hap sequence	sample number	median	Mean ± SD	Marker R^2 (%)
IB	hap1	GG/TT	101	97	96.31±5.15	10.00
	hap2	GT/TT	4	94.5	90.75±10.05	
	hap3	GG/TG	7	92	90.14±5.87	
en-I	hap1	CC/TA/CC/GA	3	25	25.00±2.00	15.93
	hap2	CC/TT/CC/GG	32	22	21.13±5.09	
	hap3	CC/TA/CC/GG	46	21	21.61±4.00	
	hap4	CT/TA/CC/GG	5	20	24.40±10.07	
	hap5	CC/TA/CA/GG	3	19	16.67±4.04	
	hap6	CC/AA/CC/GG	15	17	17.33±4.82	
	hap7	CC/TT/CA/GG	3	17	14.00±5.20	
en-OEMF	hap1	AT/CA/CA/CC	3	8	9.00±2.65	34.30
	hap2	AT/CC/CA/CA	3	4	6.00±4.36	
	hap3	AA/CC/CC/CA	25	2	3.04±2.57	
	hap4	AA/CC/CC/CC	17	2	2.59±2.45	
	hap5	AA/CC/CA/CA	6	0	0.83±1.33	
en-OEUB	hap1	GG/CG	67	12	12.03±4.67	7.44
	hap2	GG/GG	23	11	10.22±3.20	
	hap3	GA/CG	5	9	10.20±2.68	
	hap4	GA/GG	5	8	7.60±1.82	
en-TEBF	hap1	TT/AA/GG/GG/AA/TT	3	19	19.00±6.00	42.07
	hap2	AA/AA/GG/GG/AA/AA	4	12	12.25±2.06	
	hap3	TA/AA/GG/GG/AA/AA	8	11.5	10.50±3.42	
	hap4	TT/AA/GG/GG/AA/AA	4	10.5	10.75±4.27	
	hap5	TA/AA/GG/GG/AA/AT	7	10	8.57±3.95	
	hap6	TA/GA/GG/GG/AA/AA	7	10	8.43±2.76	
	hap7	TT/AA/GG/GG/AA/AT	3	10	10.67±3.06	
	hap8	TT/GA/GG/GG/AA/AT	3	10	10.67±4.04	
	hap9	AA/AA/GG/GG/AA/AT	4	9	9.25±1.26	
	hap10	AA/GG/GG/GG/AA/AA	3	9	6.33±4.62	
	hap11	AA/GA/GG/GG/AA/AA	5	8	7.40±4.16	
	hap12	TA/GA/GG/GG/AA/AT	3	8	7.67±2.52	
en-TEMF	hap1	GA/GG/CC/GG	5	8	6.60±6.54	17.57
	hap2	AA/GG/CC/GG	20	6.5	7.85±6.56	
	hap3	AA/CG/CC/GG	32	5.5	7.13±7.21	
	hap4	GA/CG/CC/GG	16	2	3.13±3.12	
	hap5	GA/CG/AC/GG	8	1	1.75±1.58	
	hap6	GG/CG/CC/GG	4	1	1.75±1.50	
	hap7	AA/CG/AC/GA	3	0	1.67±2.89	
	hap8	GA/CG/CC/GA	3	0	0.00±0.00	

en-Y	hap1	AA/AA/TG	3	24	21.33±5.51	9.99
	hap2	AA/AA/TT	7	19	19.71±5.82	
	hap3	AT/TA/TT	12	16.5	16.83±8.37	
	hap4	AATT/TT	23	15	13.91±6.43	
	hap5	AA/TA/TT	29	13	14.31±5.94	
	hap6	AT/TA/TG	3	11	14.33±10.41	
ep-TEMF	hap1	CA/AA	4	0.5	0.50±0.58	7.95
	hap2	CC/AA	64	0	0.14±0.39	
	hap3	CC/AT	26	0	0.46±0.76	
ep-Y	hap1	TT/AA	65	4	3.80±1.53	18.21
	hap2	GG/AA	3	4	4.33±1.53	
	hap3	TG/AA	6	2	2.00±1.10	
	hap4	TT/AG	8	1.5	2.00±1.51	

Table S10. The BMP-related genes having the deleterious exonic BSA-SNPs in each strain (in a separated excel file).
